# Photothermal Controlled‐Release Immunomodulatory Nanoplatform for Restoring Nerve Structure and Mechanical Nociception in Infectious Diabetic Ulcers

**DOI:** 10.1002/advs.202300339

**Published:** 2023-05-06

**Authors:** Le Jiang, Xiangyi Wu, Yifan Wang, Chunlin Liu, Yixian Wu, Jingyun Wang, Nan Xu, Zhijun He, Shuqin Wang, Hao Zhang, Xiumei Wang, Xiong Lu, Qian Tan, Xiaodan Sun

**Affiliations:** ^1^ State Key Laboratory of New Ceramics and Fine Processing School of Materials Science and Engineering Tsinghua University Beijing 100084 P. R. China; ^2^ Key Laboratory of Advanced Materials of Ministry of Education of China School of Materials Science and Engineering Tsinghua University Beijing 100084 P. R. China; ^3^ Department of Burns and Plastic Surgery Nanjing Drum Tower Hospital The Affiliated Hospital of Nanjing University Medical School No. 321, Zhongshan Road Nanjing Jiangsu 210008 China; ^4^ Key Lab of Advanced Technologies of Materials Ministry of Education School of Materials Science and Engineering Southwest Jiaotong University Chengdu Sichuan 610031 China

**Keywords:** immunomodulation, infectious diabetic ulcers, mechanical nociception, photothermal controlled‐release, polydopamine‐reduced graphene oxide

## Abstract

Infectious diabetic ulcers (IDU) require anti‐infection, angiogenesis, and nerve regeneration therapy; however, the latter has received comparatively less research attention than the former two. In particular, there have been few reports on the recovery of mechanical nociception. In this study, a photothermal controlled‐release immunomodulatory hydrogel nanoplatform is tailored for the treatment of IDU. Due to a thermal‐sensitive interaction between polydopamine‐reduced graphene oxide (pGO) and the antibiotic mupirocin, excellent antibacterial efficacy is achieved through customized release kinetics. In addition, Trem2^+^ macrophages recruited by pGO regulate collagen remodeling and restore skin adnexal structures to alter the fate of scar formation, promote angiogenesis, accompanied by the regeneration of neural networks, which ensures the recovery of mechanical nociception and may prevent the recurrence of IDU at the source. In all, a full‐stage strategy from antibacterial, immune regulation, angiogenesis, and neurogenesis to the recovery of mechanical nociception, an indispensable neural function of skin, is introduced to IDU treatment, which opens up an effective and comprehensive therapy for refractory IDU.

## Graphical abstract

In this study, a photothermal controlled‐release immunomodulatory hydrogel nanoplatform is tailored for the treatment of infectious diabetic ulcers (IDU), providing a full‐stage strategy from antibacterial, immune regulation, angiogenesis, and neurogenesis to the recovery of mechanical nociception, an indispensable neural function of skin, which opens up an effective and comprehensive therapy for refractory IDU.

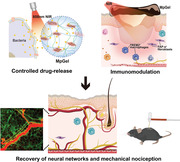

## 1 Introduction

**Figure 1:**
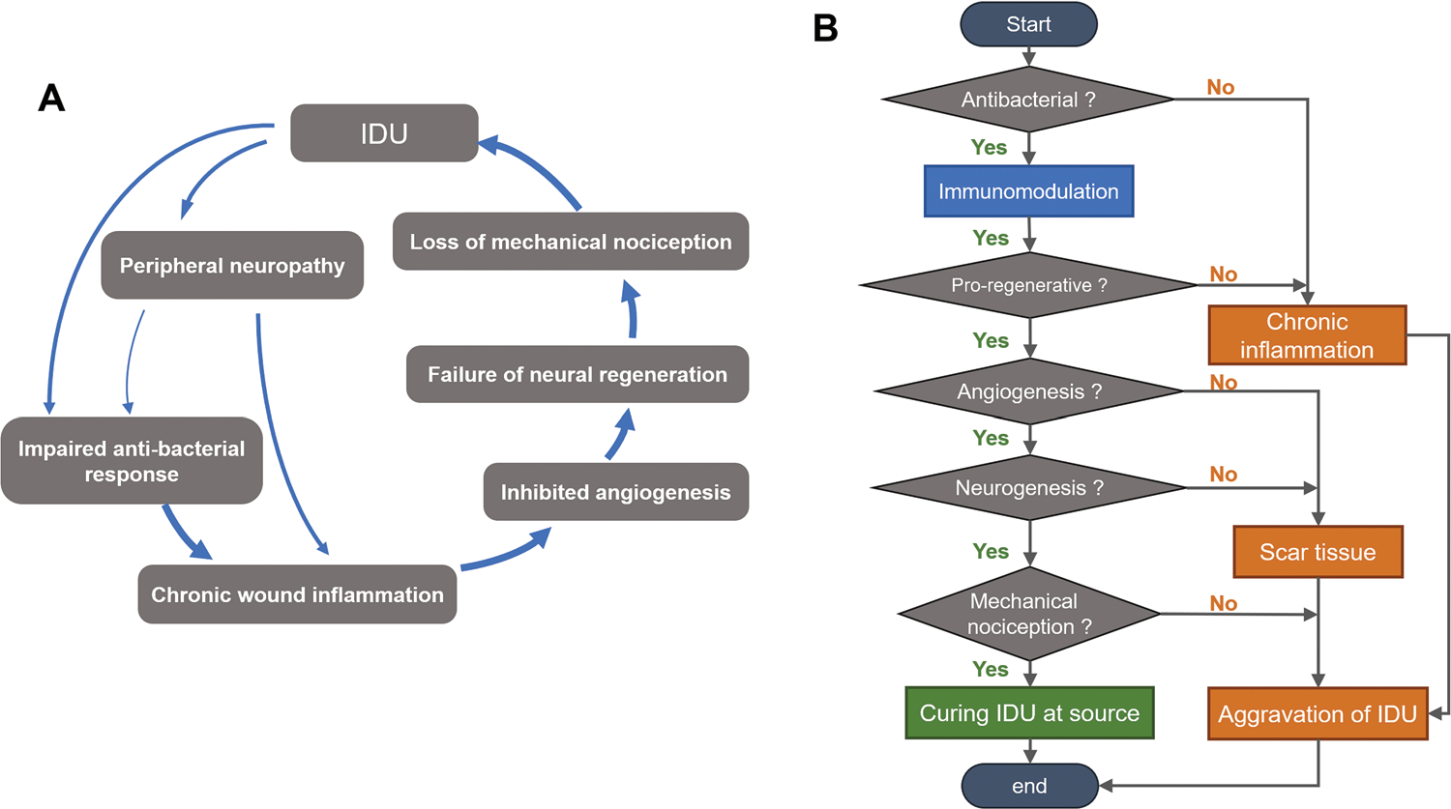
A) IDU pathological vicious circle and B) task‐oriented treatment plan.

Diabetes affects over 500 million patients worldwide.^[^ ^1^ ^]^ Refractory infectious diabetic ulcers (IDU) is the leading cause of non‐traumatic amputation in diabetes patients because it causes peripheral neuropathy, vascular disorders, and, ultimately, diabetic ulceration.^[^ ^2^, ^3^, ^4^ ^]^ In particular, peripheral neuropathy, one of the hallmarks of diabetes, leads to the loss of protective mechanical nociception so that external mechanical stresses cannot react in time, resulting in susceptibility to ulcers.^[^ ^5^, ^6^ ^]^ Moreover, it has been suggested that nociceptive nerves can detect bacteria rapidly, activating a protective immune response to combat infection and inflammation.^[^ ^7^, ^8^ ^]^ However, peripheral neuropathy associated with IDU may result in ineffective anti‐infection and anti‐inflammatory responses. Furthermore, neuropathy can impair vital cells in tissue regeneration, including fibroblasts for extracellular matrix (ECM) deposition and endothelial cells for angiogenesis.^[^ ^8^, ^9^, ^10^ ^]^ The restoration of protective mechanical nociception relies on inflammation release and blood supply. However, neuropathy‐induced impaired bacterial detection, chronic inflammation, deregulated immune response, and restricted angiogenesis impedes the repair of neural networks.^[^ ^9^, ^10^, ^11^ ^]^ Accordingly, chronic IDU might worsen due to a pathological vicious circle as shown in **Figure** **1**A.

Therefore, a rapid and synergistic strategy for achieving neural network regeneration and mechanical nociception for IDU treatment is required to break this vicious cycle. Figure 1B depicts that IDU‐related pathological changes require a well‐organized treatment plan. Since diabetes impairs the immune system's normal antimicrobial response, eliminating infection is a priority in initiating treatment.^[^ ^9^, ^12^ ^]^ The infection can prolong pathogenic inflammation and cause wound chronicity, gangrene, or sepsis if not treated properly.^[^ ^12^ ^]^ Immunomodulation, the second phase of IDU healing, focuses on the recruitment of and expression of pro‐regenerative immune cells and cytokines. In IDU, the dysregulated immune response to the wound leads to a chronic inflammatory state, where the impaired transition of pro‐ (M1) to anti‐inflammatory (M2) macrophages prevents progression to the proliferative phase, and impedes tissue regeneration processes, including angiogenesis, ECM remodeling, and re‐epithelialization.^[^ ^5^, ^13^, ^14^, ^15^, ^16^ ^]^ Therefore, proper immune system regulation is a pre‐requisite for the regeneration stage. In the next stage, angiogenesis provides nutrients for wound healing.^[^ ^5^ ^]^ Failure to stimulate angiogenesis leads to metabolic disorders, especially in diabetic wounds.^[^ ^5^ ^]^ In addition, blood vessels play a critical role in developing a restorative neurovascular, which is intended to induce nerve fiber sprouting.^[^ ^17^ ^]^ Later, the development of an associated neural structure follows the regeneration of the vascular bed. If the responsible neural network recovery fails, neuropathic nerve residues will not restore the mechanical nociception.^[^ ^18^ ^]^ In summary, successful IDU wound healing requires the completion of all tasks.

Antibiotics are the most prevalent antibacterial agents in clinical practices.^[^ ^19^, ^20^ ^]^ However, intravenous antibiotics can produce systemic side effects. Hydrogel wound dressings loaded with antibiotics have recently been used as sustained‐release agents. Fan et al. designed a hydrogel dressing that sustainably releases antibiotics by encapsulating ciprofloxacin (CIP) in a hydrogel.^[^ ^21^ ^]^ However, continuous administration of sustained‐release antibiotics might induce acquired drug resistance, leading to treatment failure.^[^ ^22^, ^23^ ^]^ Gao et al. combined polydopamine (PDA) nanoparticles with CIP through thermosensitive interaction and loaded them into hydrogel wound dressings.^[^ ^24^ ^]^ Once near‐infrared (NIR) light is applied, PDA nanoparticles generate a large amount of heat through the photothermal effect, releasing CIP.^[^ ^24^ ^]^ Photothermal response controls CIP release in this drug release system, achieving antibacterial efficacy.^[^ ^24^ ^]^ Furthermore, photothermal nanoparticles can promote the photothermal controlled release and improve cell membrane permeability when the boost releases antibiotics. Nanoparticle photoporation, based on photothermal nanoparticles such as graphene family and Au nanoparticles, is a novel and efficient method for the intracellular delivery of biological agents, which has the potential to improve the bacterial intracellular delivery of antibiotics.^[^ ^25^ ^]^ However, potential nanotoxicity prevents its clinical applicability since it relies on nanoparticle‐cell contact.^[^ ^25^ ^]^ Xiong et al. embedded photothermal iron oxide nanoparticles into biocompatible electrospun nanofibers, avoiding direct contact between nanoparticles and cells and inducing cell membrane permeability through the photothermal effect, promoting the delivery of effector molecules into cells.^[^ ^25^ ^]^

Therapeutic angiogenic drugs commonly used in clinical practice have short‐term angiogenic effects, such that blood vessels regress with the depletion of angiogenic agents.^[^ ^17^ ^]^ Vascular Endothelial Growth Factor (VEGF), the most common angiogenic agent, promotes infection and inflammation, which hinders infectious wound healing.^[^ ^26^ ^]^ On the other hand, angiogenesis may be accelerated by inducing an appropriate immune response,^[^ ^27^, ^28^ ^]^ increasing long‐term vascular regeneration, and reducing the risk of VEGF‐induced infection. However, common pharmacological or biological immunomodulators can cause systemic immunological disorders. In recent years, nanomaterial‐based immunomodulation has attracted considerable attention because of its high efficacy and safety.^[^ ^29^ ^]^ Tu et al. prepared a PDA‐reduced graphene oxide (pGO) composite hydrogel.^[^ ^30^ ^]^ The results demonstrated that pGO activated the polarization of macrophages to the M2 phenotype and the angiogenesis of endothelial cells via a paracrine mechanism, promoting the repair of diabetic ulcers.^[^ ^30^ ^]^ The treatment of IDU from the perspective of bacterial clearance and vascular regeneration has been frequently reported,^[^ ^5^, ^31^, ^32^, ^33^, ^34^ ^]^ whereas nerve regeneration has received little attention. In particular, there have been few reports on the recovery of mechanical nociception yet.^[^ ^35^ ^]^

This study aims to design a photothermal controlled‐release immunomodulatory hydrogel nanoplatform based on the thermosensitive interaction between pGO and the antibiotic mupirocin (Mup), to achieve effective bacterial clearance through photothermal controlled‐release, which is the base of regeneration, and to induce a suitable immune microenvironment through immunomodulation to promote collagen remodeling and vascular regeneration, thus achieving the recovery of accompanied neural network and mechanical nociception. Full‐stage strategies from antibacterial, immune regulation, angiogenesis, and neurogenesis, to the recovery of mechanical nociception, an indispensable neural function of skin, are urgently to be introduced to IDU treatment.

## 2 Results and Discussion

### 2.1 Characterization of pGel

The pGO was obtained as described in the Methods section of Supporting Information. According to the nitrogen distribution images, the functionalized catechol groups were spread uniformly over the surface of the pGO, which had a wrinkly, translucent sheet shape (Figure S1, Supporting Information). The reduction of pGO was analyzed, and the carbon and oxygen 1s spectra revealed that more than half of the GO was successfully reduced by PDA (Figure S2 and Tables S1 and S2, Supporting Information). Further, a hydrogel wound dressing incorporated with pGO was prepared (denoted as pGel) based on our previously reported chitosan‐polyolefin network hydrogel.^[^ ^36^ ^]^ The SEM images demonstrated that the pGel had a typical porous network structure of the hydrogel (Figure S3, Supporting Information). The tensile strength of pGels with different proportions of pGO was tested (Figure S4, Supporting Information), and the pGel with 5 wt% pGO showed a skin‐similar Young's modulus (≈30 kPa). Such a biomimetic mechanical property satisfies the requirement for skin dressing.^[^ ^37^ ^]^ Furthermore, the adhesion performance of pGel was tested, and it displayed extraordinary adhesiveness to various solid surfaces, including different organs (Figure S5, Supporting Information). And the live–death test of Schwann cells showed no cytotoxicity of pGel (Figure S6B, Supporting Information).

### 2.2 Mup Release Kinetics Differentiating Antibacterial Properties

**Figure 2:**
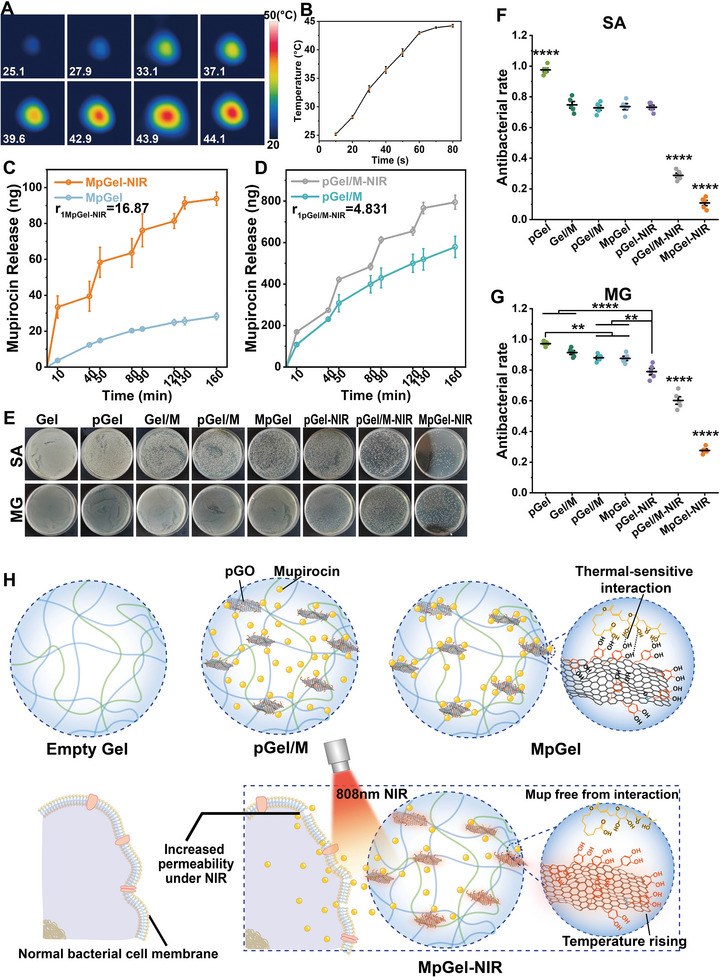
Mup release kinetics differentiating antibacterial properties. A) Photothermal response characterization of pGel using an infrared camera. B) A plot of temperature variation over time in the photothermal process. C,D) Mupirocin release rate over time range 0–160 min of MpGel‐NIR (orange), MpGel (light blue), pGel/M‐NIR (gray), and pGel/M (light green), defining r1 as the slope of 0–10 min (laser on) divided by that of 10–40 min (laser off) for the NIR‐treated groups. E–G) Antibacterial test in vitro using the spread plate method for Gel, pGel, Gel/M, pGel/M, MpGel, pGel‐NIR, pGel/M‐NIR, and MpGel‐NIR with *Staphylococcus aureus* (SA) and *Escherichia coli* (MG1655) bacteria solution (E), and the quantified antibacterial rate of SA (F) and MG (G) (*n* = 5). Each dot in the plots in (F) and (G) represents one animal, and the *p* values are determined using one‐way ANOVA and Tukey's post hoc test. **p < 0.01, ****p < 0.0001. H) Schematic diagram of Empty Gel, pGel/M, and MpGel, and the mechanism of controlled‐release of Mup in MpGel‐NIR system, where Mup is released from the thermal‐sensitive interaction when pGO generates heat under NIR. Meanwhile, the photothermal response of pGO enhances bacterial cell membrane permeability, leading to the entry of Mup into bacteria. The dual function of pGO significantly improves the antibacterial effect of the MpGel‐NIR system.

Reduced graphene oxide (rGO) has become a preferred drug‐loading nanoplatform because of its large surface area, rich surface chemistry, and excellent photothermal responsiveness.^[^ ^38^, ^39^ ^]^ Furthermore, pGO exhibits better biocompatibility, adhesivity, and stable dispersion in aqueous solutions than rGO owing to the mussel‐like nature of PDA.^[^ ^40^, ^41^, ^42^ ^]^ The photothermal response of the pGel was verified to increase by 20 °C in 80 s, with the highest temperature of around 44 °C (**Figure** **2**A,B), which is not expected to harm normal tissues,^[^ ^36^ ^]^ and the CCK8 results indicated no significant influence on cell viability during 3 min of NIR treatment (Figure S6A, Supporting Information). pGO is a 2D material with large specific surface area, and the modified dopamine provides a large number of binding sites for the loading of Mup. Herein, pGO was loaded with Mup via thermosensitive interactions to obtain MpGO in advance, which was added to the hydrogel monomer for mixing (Figure S2 and Tables S1 and S2, Supporting Information) and cross‐linking to obtain the MpGO nanocomposite hydrogel (denoted as MpGel). In contrast, Mup was loosely dispersed in the hydrogel (denoted as pGel/M) without interacting with pGO in the drug carrier system generated by mixing pGO and Mup directly into the hydrogel (Figure 2H). The release kinetics of the two integration modes (pGel/M and MpGel) were investigated. The initial release amount (the first 40‐min interval) of pGel/M was over ten times that of MpGel (Figure 2C,D). The MpGel‐NIR system (MpGel treated by repeated 808‐nm NIR with every 10 min on and every 40 min off) displayed a controlled on–off switch release of Mup. When NIR was on, drug release increased significantly, but when NIR was off, it was negligible (Figure 2C). For the MpGel, when NIR was on, pGO produced heat because of its photothermal property (Figure 2A,B), and the thermal‐sensitive interaction between Mup and pGO was disrupted when the temperature rose, releasing Mup molecules from the interaction and creating a NIR‐controlled Mup‐release switch (Figure 2H). In contrast, the pGel/M system passively released Mup driven by the diffusion effect, resulting in a temporal burst release in the initial stage (Figure 2D, plot of pGel/M), which was not conducive to sustained release. To better highlight the photothermal‐responsive release capabilities of MpGel, the coefficient of photothermal‐responsive release was determined as *r* = photothermal release rate/pre‐photothermal release rate. For the first round of NIR, *r* of the MpGel‐NIR was 3.5 times that of pGel/M‐NIR (Figure 2C,D), indicating that although temperature increase can promote diffusion in pGel/M‐NIR, it is far from the effectively controlled release by the switch of NIR. Therefore, compared to pGel/M‐NIR, a better‐controlled drug release capacity of MpGel‐NIR was achieved.

**Figure 3:**
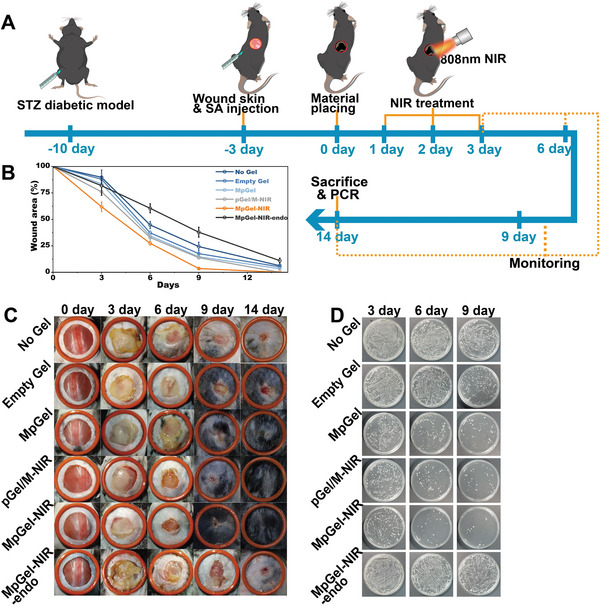
Resurfacing of skin tissue and antibacterial effects in vivo. A) Experimental timeline for the antibacterial and wound‐healing experiments. Skin wound was generated 7 days after streptozotocin (STZ) injection, and mice were treated with one of the following treatments: No Gel, Empty Gel, Gel/M, pGel, pGel‐NIR, pGel/M, MpGel, pGel/M‐NIR, MpGel‐NIR, MpGel‐NIR‐endo. NIR treatment on Days 1, 2, and 3 used 808 nm NIR irradiation. Mice were monitored for wound area and antibiotic activity (illustrated by orange dotted line) on Days 3, 6, 9, and 14 after material treatment. B–D) Plotted quantification (*n* = 3) (B) and photograph (C) of the skin wound area on Days 0, 3, 6, 9, and 14, and antibiotic activity (D) on Days 3, 6, and 9 of No Gel, Empty Gel, MpGel, pGel/M‐NIR, MpGel‐NIR, and MpGel‐NIR‐endo treated groups.

To investigate the antibacterial effects of different drug release kinetics, hydrogel platforms were used in vitro and in vivo. Gram‐positive *Staphylococcus aureus* (SA) and Gram‐negative MG1655 were investigated in vitro (Figure 2E–G). For the in vivo study, the as‐prepared hydrogels were administered to a 1 cm^2^ streptozotocin diabetic defect model infected with SA (Figure 1A). Gel and pGel were demonstrated to have negligible anti‐infection effects in vitro and in vivo (Figures 2E and **3**D). Although Gel/M had a slight antibacterial action, short‐term use of large quantities of Mup might lead to acquired resistance to bacteria in vitro and in vivo (Figures 2E and 3D and Figure S7, Supporting Information). Chronic administration of antibiotics in IDU can cause acquired drug resistance,^[^ ^43^, ^44^, ^45^ ^]^ which could be a contributing factor to Gel/M repair failure (Figure S8, Supporting Information). Therefore, instead of the sustained Mup release, customized Mup release kinetics was proposed to optimize the antibacterial performance. It can be seen from Figure 3D that the bacteria in MpGel‐NIR group on Day 6 and 9 is significantly less than that in other groups. The MpGel‐NIR group had the best antibacterial performance in vivo (Figure 3D and Figure S7, Supporting Information) and in vitro (Figure 2E–G). Importantly, compared with pGel/M‐NIR, MpGel‐NIR treatment prevented drug resistance due to continuous exposure of bacteria to the drug environment. Furthermore, photothermal treatment can improve cell membrane permeability and intracellular drug delivery.^[^ ^25^, ^46^ ^]^ This suggests that MpGel‐NIR could improve antibiotic release to bacteria with highly permeable membranes when NIR was on, as illustrated in the schematic diagram in Figure 2H, which maximized antimicrobial efficacy through the synergistic action of antibiotics and photothermal treatment. The excellent antibacterial performance of MpGel‐NIR removes barriers to IDU wound repair.

### 2.3 MpGel Prevents Scar Formation by Recruiting Trem2^+^ Macrophages and FAP‐*α* ^+^ Fibroblasts

Scar tissue forms rapidly following traumatic injury to provide a basic barrier, but it does not regenerate adnexal structures or restore sophisticated tissue functions, making the scar vulnerable to a second injury which is detrimental in the IDU situation, especially. Therefore, research and clinical practice in biomedical material engineering focus on rapid barrier restoration without scar formation. Regulating macrophages and fibroblasts, which play crucial roles in skin tissue remodeling,^[^ ^18^ ^]^ is expected to alter the fate of scar formation. Macrophages, as key elements in the innate immune system, participate in all stages of the injury response, from the acute wound inflammatory response to tissue remodeling.^[^ ^47^ ^]^

Recent investigations have provided insights into the mechanistic aspects of diabetic ulcer pathogenesis that deregulate the immune response, in which improper recruitment, impaired activation, and survival of macrophages contributed to the stalled non‐healing state.^[^ ^10^, ^48^ ^]^ Moreover, the impaired transition of proinflammatory (M1) to pro‐regenerative (M2) macrophages in IDU may cause a perturbed, ineffective inflammatory response, which is likely the most dysregulated phase of diabetic wound healing.^[^ ^5^, ^13^, ^14^, ^15^, ^16^ ^]^

**Figure 4:**
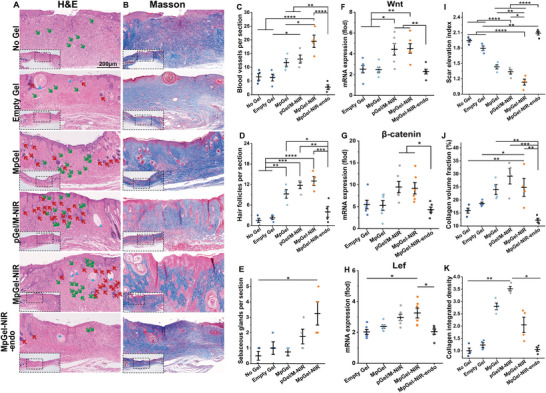
Histopathological analysis of scar formation. A) H&E and B) Masson's trichrome staining of the wound skin of the No Gel, Empty Gel, MpGel, pGel/M‐NIR, MpGel‐NIR, and MpGel‐NIR‐endo treated groups on Day 14 (blood vessels, hair follicles, and sebaceous glands are indicated by green arrows, red arrows, and blue triangles, respectively). C–E) The number of blood vessels (C), hair follicles (D), and sebaceous glands (E) per section (*n* = 4). F–H) Relative mRNA expression of Wnt (F), *β*‐catenin (G), and Lef (H), (*n* = 6). I–K) Quantitative assessment of collagen remodeling by scar elevation index (I), collagen volume fraction (J), and collagen integrated density (K) (*n* = 4). Each dot in the plots represents a single animal; *p* values in (C), (D), (F), (G), (I) and (J) are determined using one‐way ANOVA and Tukey's post hoc test; the *p* values in (E), (H), and (K) are determined by nonparametric test using Kruskal–Wallis and all pairwise for multiple comparisons, *p < 0.05, **p < 0.01, ***p < 0.001, and ****p < 0.0001.

The reducibility and antioxidation of reduced GO (including pGO) increase the M2 ratio at the wound,^[^ ^49^, ^50^, ^51^ ^]^ preventing fibrosis and promoting skin tissue remodeling.^[^ ^52^, ^53^ ^]^ The physicochemical characteristics and surface functionalization of carbon nanomaterials significantly alter their inflammatory properties when applied to wound healing.^[^ ^54^ ^]^ Specifically, dopamine has anti‐inflammatory and M2 polarization‐promoting effects and is frequently employed as a graphene material surface modifier for skin tissue regeneration.^[^ ^55^, ^56^, ^57^ ^]^ Recently, it has been reported that PDA, because of its catechol group, exhibits immunomodulatory properties in the skin regeneration of IDU.^[^ ^42^ ^]^ Therefore, pGO is a promising immunomodulator for anti‐inflammatory and scar‐prevention treatments. In the No Gel group, the wound area assessment, hematoxylin and eosin (H&E) and Masson's trichrome staining revealed an unclosed wound surrounded by typical scar tissue (Figures 3B,C and **4**A). The Empty Gel, Gel/M, pGel, pGel/M, and pGel‐NIR also showed unclosed wounds at Day 14 (Figure 3B,C and Figure S8, Supporting Information). Furthermore, it can be seen from Figure 3B,C that the wound treated by MpGel‐NIR recovers significantly faster on Day 6 and 9, and removes wound inflammation more quickly (yellow bacterial membrane) compared to pGel/M‐NIR. Despite a few hair follicles in these groups (Figure 4A and Figure S9A, Supporting Information), they displayed horizontally aligned accumulation of collagen fibers (Figure 4B and Figure S9A, Supporting Information), indicating progressive fibrosis. More specifically, No Gel and Empty Gel groups demonstrated significantly lower level of collagen volume fraction and integrated density (Figure 4J,K), suggesting the ECM degradation and thus insufficient sites for collagen deposition. Although pGel, Gel/M, pGel/M, pGel‐NIR, and pGel/M‐NIR gained high collagen fraction (Figure 4J), the high scar elevation indices (SEI) (Figure 4I) indicated the potential scar hypertrophy. The MpGel‐NIR treated group had the fastest wound closure speed (Figure 3B). It also exhibited a clear regenerated tissue with a physiologically undulated surface, regeneration of hair follicles, and epidermal cysts (Figure 4A,D,E) which represent the initial site of neogenic hair follicles.^[^ ^29^ ^]^ It also featured with “basket‐weave” like collagen fibers resembling the physiologic dermal collagen architecture of murine skin and SEI that is close to 1 and significantly lower than the others (Figure 4B,I). Some regenerated hair follicles developed into new sebaceous glands in the thicker dermis of the MpGel‐NIR groups (Figure 4A–E). All these results suggested a regenerative progression in MpGel‐NIR group. After 4 weeks of treatment, pGO‐treated wound sites had a more complex structure and resembled normal skin (Figure S11, Supporting Information). Furthermore, qRT‐PCR results demonstrated that MpGel‐NIR group significantly activated the Wnt/*β*‐catenin signal axis (Figure 4F–H and Figure S12, Supporting Information), which regulates skin tissue regeneration.^[^ ^58^, ^59^, ^60^ ^]^ Histological and molecular results indicated that either pGel itself or with NIR application could only generate slight reparative effects; although introducing Mup was expected to clear bacteria to achieve regenerative environment, inappropriate modes of drug release (Gel/M, pGel/M, and pGel/M‐NIR) can also lead to unsatisfactory results. In all, MpGel‐NIR reduced bacteria, accelerated wound closure, and prevented scar formation, providing a histological structure for functional recovery.

**Figure 5:**
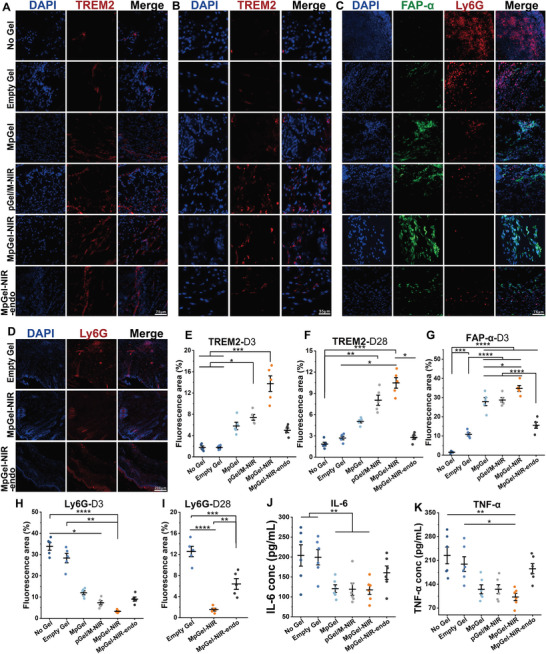
Recruitment of Trem2^+^ macrophages, FAPa^+^ fibroblasts, and neutrophils, and expression of inflammatory factors in wounds. Fluorescent images of Trem2^+^ macrophages (TREM2) in the wound on A) Day 3 and B) 28 after material treatment and E,F) relative quantifications (*n* = 5). Fluorescent images of FAP‐*α* ^+^ fibroblasts (FAP‐*α*) and neutrophils (Ly6G) on C) Day 3 and D) 28 following material treatment and G–I) relative quantifications (*n* = 5). J) IL‐6 and K) TNF‐*α* concentrations determined by ELISA. Each dot in the plots represents a single animal, and *p* values in (G), (I), and (J) are determined using one‐way ANOVA with Tukey post hoc test; whereas *p* values in the remaining plots are determined by nonparametric test using Kruskal–Wallis and all pairwise for multiple comparisons: *p < 0.05, **p < 0.01, ***p < 0.001, ****p < 0.0001.

The anti‐inflammatory effect of pGO supported this scar inhibition under NIR treatment, as shown in the immunofluorescence results of neutrophils (Ly6G) on Days 3 and 28 (**Figure** **5**C,D,H,I and Figure S13B,C, Supporting Information) and the ELISA results of IL‐6 and TNF‐*α* on Day 3 (Figure 5J,K and Figure S13D,E, Supporting Information), where MpGel‐NIR statistically reduced the number of inflammatory cells and the expression of inflammatory cytokines. Although the anti‐inflammatory cytokines suggest a pro‐regenerative environment where macrophages are prone to M2 polarization, the benefits of M2 on scars are not guaranteed, especially considering its activation on fibroblasts.^[^ ^61^ ^]^ After an injury, macrophage signals affect the proliferation, migration, and activation of fibroblasts, resulting in varying rates, amounts, and arrangements of collagen deposition.^[^ ^28^, ^29^, ^62^ ^]^ With the advancement of single‐cell sequencing technology, the classification of macrophages is no longer limited to M1 and M2, but classified into subpopulations according to function or gene expression, allowing for a more precise understanding of their activity. Therefore, instead of detecting the M2 phenotype, it is vital to also ensure that the beneficial subpopulation is recruited. Trem2^+^ macrophages promote bacterial clearance, collagen remodeling, and full‐thickness skin regeneration during wound healing.^[^ ^28^, ^63^ ^]^ Here, it was demonstrated that the recruitment of Trem2^+^ macrophages on Day 3 was statistically higher in the pGel/M‐NIR and MpGel‐NIR groups, and the MpGel‐NIR group maintained this increase on Day 28, indicating the beneficial recruitment of macrophages by pGO. (Figure 5A,B,E,F and Figure S13, Supporting Information).

In contrast, FAP‐a^+^ fibroblasts regulate the deposition of the early temporary matrix during skin wound healing.^[^ ^64^ ^]^ Our results demonstrated that pGel/M‐NIR and MpGel‐NIR increased the early recruitment of FAP‐a^+^ fibroblasts (Day 3, Figure 5C,G and Figure S13, Supporting Information). The quantification of FAP‐a^+^ fibroblasts on Day 3 was consistent with the remodeling complexity of collagen architecture in Masson's trichrome staining, where the pGel/M‐NIR and MpGel‐NIR groups displayed the “basket weave” collagen fiber pattern, proper collagen volume fraction, and SEI close to 1 instead of the insufficient deposition or over‐deposition characterized by horizontally oriented collagen bundles and higher SEI value observed in the other groups (Figure 4B and Figures S9 and S10, Supporting Information). The potential mechanism of recruitment or activation of FAP‐*α* ^+^ fibroblasts involves crosstalk with immune cells.^[^ ^65^, ^66^ ^]^

### 2.4 MpGel‐NIR Results in Vascularization

**Figure 6:**
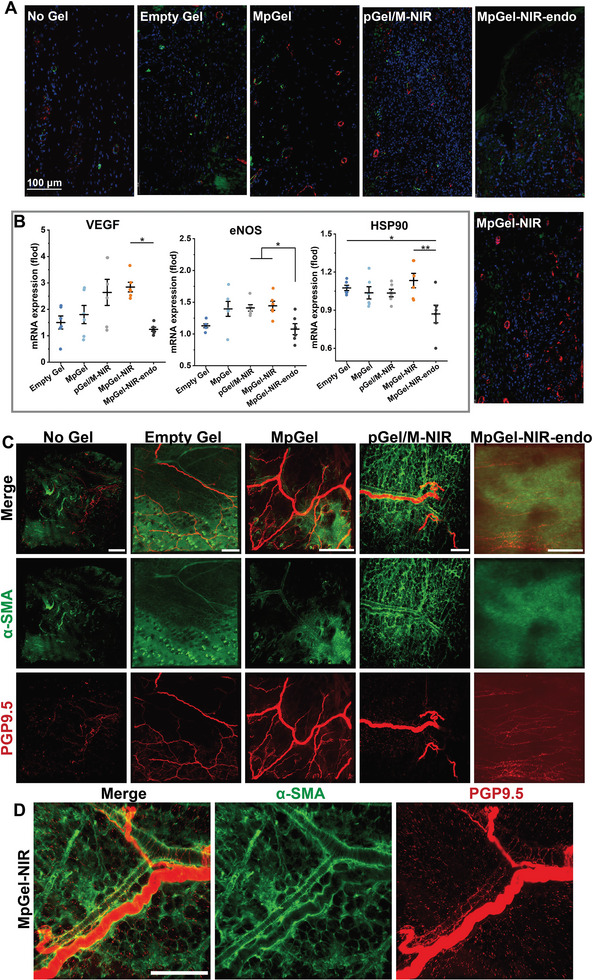
Characterization of vascularization. A) Fluorescence images of sections with blood vessel endothelial cells (CD31) in green and pericytes in mature blood vessels (*α*‐SMA) in red on Day 14. B) Plots of relative mRNA expression of VEGF, eNOS, and HSP90 (*n* = 6). Each dot in the plots representing one animal and *p* values in eNOS and HSP90 are determined by one‐way ANOVA with Tukey post hoc test while in VEGF, determined by nonparametric test using Kruskal–Wallis and all pairwise for multiple comparisons; *p < 0.05, **p < 0.01, ***p < 0.001. C,D) Fluorescent images of cleared wound skin tissue with blood vessels (*α*‐SMA) in green and nerves (PGP9.5) in red after 4 weeks, treated with No Gel, Empty Gel, MpGel, pGel/M‐NIR, and MpGel‐NIR‐endo (C) and MpGel‐NIR (D), and all the scale bars represent 200 µm.

The recruitment of Trem2^+^ macrophages regulates collagen remodeling and promotes angiogenesis.^[^ ^28^ ^]^ Damaged endothelial progenitor cells and low cytokines limit angiogenesis in diabetic wounds.^[^ ^67^ ^]^ Moreover, pathological angiogenesis can result in functional ischemic wounds.^[^ ^68^ ^]^ Clinically used therapeutic angiogenic agents generate only short‐term angiogenic effects.^[^ ^17^ ^]^ With the depletion of angiogenic agents, the blood vessels regress. In addition, VEGF, the most common angiogenic agent, increases the risk of infection, inflammation, and fibrosis under specific conditions, which are detrimental to the healing of infectious wounds.^[^ ^26^, ^69^ ^]^ In contrast, the proangiogenic effect regulated by Trem2^+^ macrophages provides a sustained and comprehensive treatment for wound healing.^[^ ^28^ ^]^ These results demonstrated that pGO could effectively recruit Trem2^+^ macrophages. To determine whether immunomodulation induced angiogenesis, evaluations were performed 2 and 4 weeks after treatment, representing the vascular maturation and the wound tissue and function recovery stages of IDU repair, respectively. After 2 weeks, although the No Gel and Empty Gel displayed vessel sprouting, the MpGel‐NIR group showed a significantly increased number of blood vessels and extent of vascular infiltration at wound sites than the other groups (Figure 4A,C and Figure S9A,B, Supporting Information). In addition, the angiogenic transcriptional activity of endothelial cells was examined to better comprehend the function of the new blood vessels. The qPCR results of skin tissues at 2 weeks indicated that pGel/M‐NIR and MpGel‐NIR promoted the expression of the VEGF‐eNOS signaling axis (**Figure** **6**F–H and Figure S14B, Supporting Information), suggesting an enhanced pro‐angiogenic effect. Furthermore, Pearson's correlation analysis was performed to verify the correlation between early‐stage recruitment of Trem2^+^ macrophages and angiogenesis (Figure S15, Supporting Information). The results showed that angiogenesis was associated with the effect of Trem2^+^ macrophages, with 86.4% confidence.

Skin repair often fails due to a lack of functional mature blood vessels; immature blood vessels lack pericytes that express *α*‐smooth muscle actin (*α*‐SMA), which influences vascular contractility.^[^ ^70^, ^71^ ^]^ Here, we tested whether the endothelial marker (CD31) was co‐located with *α*‐SMA to measure blood vessel maturation. In the MpGel, pGel/M‐NIR,, and MpGel‐NIR groups, blood vessels were covered with *α*‐SMA pericytes, with MpGel‐NIR showing the highest covered ratio, suggesting the highest level of vessel maturation and the best angiogenetic effects (Figure 6A and Figure S14A, Supporting Information). Immunofluorescent labeling of optical‐cleared samples showed mature blood vessel networks in the repaired wound area after 4 weeks (Figure 6C,D, and Figure S14C, Supporting Information), except in the No Gel and Empty Gel groups. In contrast, MpGel‐NIR and pGel/M‐NIR, especially the MpGel‐NIR group, had vessels with much larger diameters and more complete networks, consistent with the higher survival of Trem2^+^ macrophages on Day 28 (Figure 5B,F). Therefore, MpGel‐NIR inhibited infection and scarring and recruited Trem2^+^ macrophages with vascularizing effects at an early stage, thereby promoting angiogenesis and vessel maturation. Indeed, inhibiting infection and scarring also paved the way for angiogenesis.

### 2.5 Regenerated Skin Neural Networks Growing along Blood Vessels

**Figure 7:**
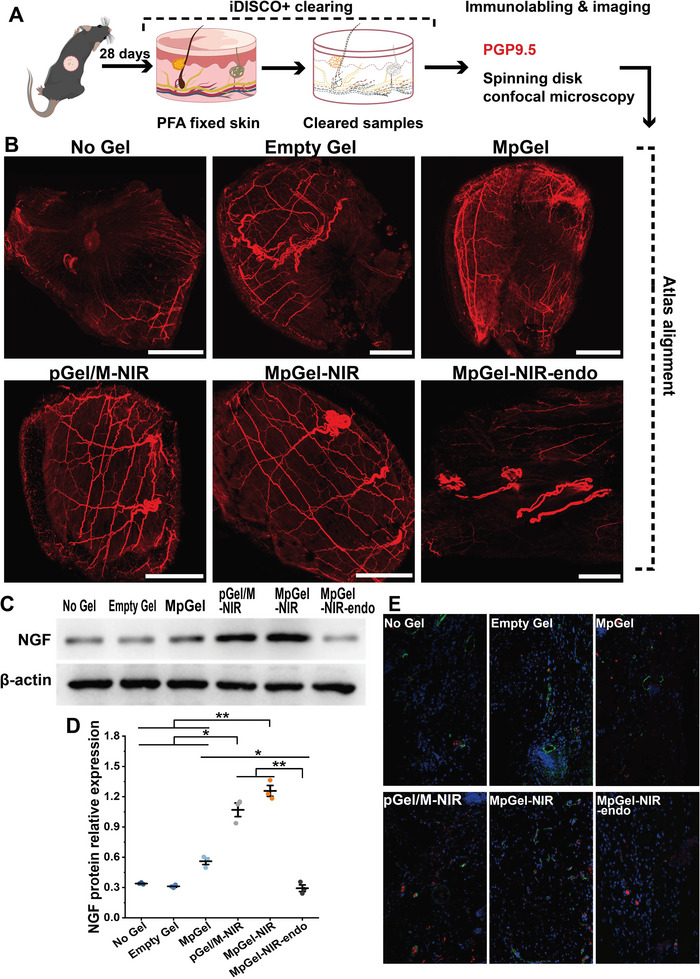
Restoration of neural networks. A) Scheme of skin tissue iDISCO^+^ clearing and imaging. B) Fluorescent images of bulk wound skin tissues displaying the nerves system (PGP9.5) in red, with all the scale bars representing 2 mm. C–E) Western‐blot of NGF (E) and the quantification of relative expression (*n* = 3); *p* values in (E) are determined using one‐way ANOVA with Games–Howell post hoc test;*p < 0.05, **p < 0.01. E) Fluorescence images of sections with blood vessel endothelial cells (CD31) in green and nerves (PGP9.5) in red.

Anti‐infection, scar prevention, and angiogenesis provide optimal conditions for IDU treatment rather than specific destinations. As discussed in the Section 1, ensuring the integrity of neural networks after skin defects is crucial for treating IDU. Thus, we hypothesized that reconstructing a dense and well‐structured vascular bed at wound sites could induce subsequent neural network reconstruction.^[^ ^72^ ^]^ Three assessments were performed from different perspectives to investigate the consequences of regeneration on neural networks. PGP9.5 was used to label skin nerve fibers, and a spinning‐disk confocal microscope was used to conduct immunolabeling‐enabled 3D fluorescence imaging of the nerve fiber network in the skin tissue after optical‐cleared tissue treatment (iDISCO, **Figure** **7**A). Visual imaging of the neural networks showed that MpGel‐NIR and pGel/M‐NIR almost completely reconstructed skin nerve fiber networks across the whole wound area, while the other groups, had a nerve fiber prohibited area at the wound center (Figure 7B and Figure S16, Supporting Information). Other groups had thinner regenerated nerve fibers than the MpGel‐NIR and pGel/M‐NIR groups (Figure 7B and Figure S16 and Video S1, Supporting Information). Second, western blotting demonstrated that MpGel‐NIR‐treated groups had statistically higher nerve growth factor (NGF) protein expression (Figure 7C,D). The results of nerve regeneration in the different groups showed a trend consistent with that of vascular network regeneration. Third, to explore the association between vascular and neural networks, blood vessel (CD31 or *α*‐SMA) and nerve fiber (PGP9.5) markers were co‐stained in histological sections (Figure 7E) and cleared wound skin tissue (Figure 6C,D) at the wound areas. Histological sections revealed that MpGel‐NIR and pGel/M‐NIR groups were characterized by co‐localized CD31 and PGP9.5, and almost all the regenerated nerve fibers were in contact with vessels in MpGel‐NIR (Figure 7E). As for the co‐staining in cleared skin tissue, the MpGel‐NIR group had the closest association between the vasculature and nerve fibers (Figure 6C,D and Figure S14C, Supporting Information), and the 3D image showed that the course of nerves was approximately concomitant to the blood vessels (Video S1, Supporting Information). However, incomplete regenerated vascular networks or relatively smaller vessel diameters in other groups resulted in either inhibited or fragile nerves.

To further prove that the observed neural network reconstruction was due to revascularization of the defect area, endostatin was administered to block angiogenesis at 2 or 4 weeks before MpGel‐NIR treatment. Blocking the VEGF‐eNOS signaling axis (Figure 6B), maturation of blood vessels (Figure 6A), and formation of vascular networks (Figure 6C,D, and Figures S9 and S14, Supporting Information) inhibited the formation of the neural networks (Figures 6C and 7B). These results indicated that blood vessels established a reparative neurovascular niche and leaded to neural network reconstruction in the wound area, similar to tissue development under normal physiological conditions. Furthermore, MpGel‐NIR‐endo blocked the vascular regeneration and the accompanied neural network reconstruction, increased the level of wound inflammatory factors (Figure 5J,K), inhibited the positive immune regulation (Figure 5A,B), and promoted the entry into a vicious circle, leading to wound healing failure (Figure 3B,C).

### 2.6 Recovery of Mechanical Nociception in Regenerated Skin

The skin, the largest organ of the human body, performs multiple functions, including barrier function, secretion, excretion, nociception, and other sensory activities. The barrier function of the skin ensures its role as the first line of defense in the human body, which is determined by the integrity, thickness, and tissue structure of the skin.^[^ ^73^ ^]^ IDU compromises normal skin barrier function. Long‐term impairment of the skin barrier function endangers life safety. Humans benefit from the timely reconstruction of skin with a similar structure comparable to that of normal skin tissue. MpGel‐NIR effectively promoted the rapid closure of IDU and regenerated skin tissue with a neurovascular structure similar to that of normal skin tissue (Figures 4A and 6D).

The primary function of the sebaceous gland is to secrete sebum to lubricate the skin and hair, preventing dryness.^[^ ^74^ ^]^ Meanwhile, sebum combines with moisture from sweat glands, cuticles, and various substances, forming an acidic sebaceous gland membrane that inhibits bacteria and fungi.^[^ ^74^ ^]^ As shown in Figure 4D,E, the MpGel‐NIR group had a statistically significant increase in hair follicles and sebaceous glands, which was conducive to the sebum secretion of skin tissue and internal bacteriostasis.

**Figure 8:**
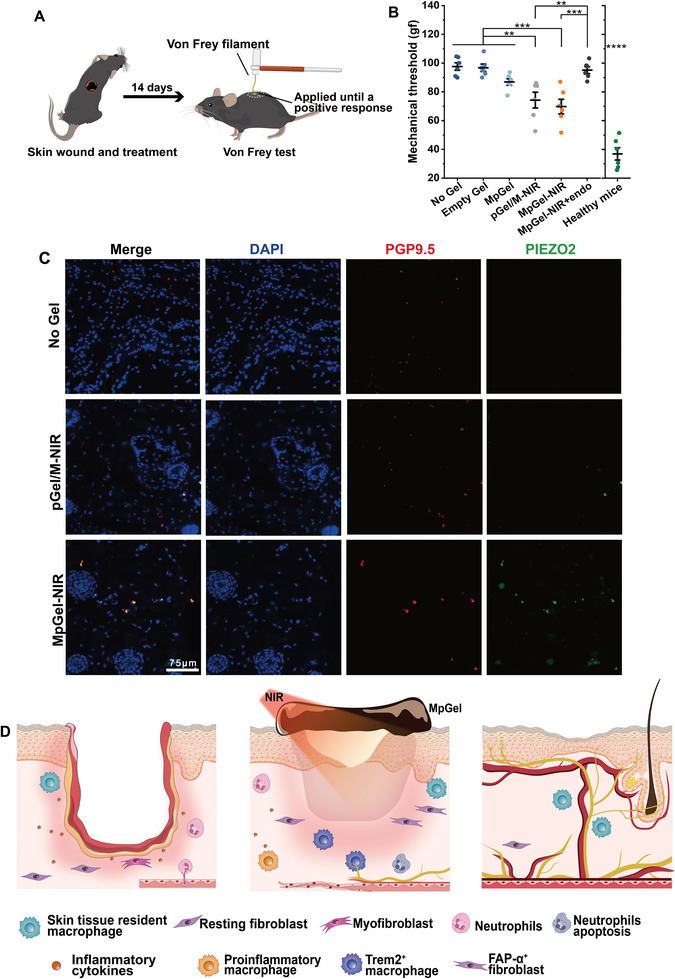
Blood vessels promote the reconstruction of nerve structure and function. A,B) Scheme of Von Frey test (A) and quantification of mechanical threshold (B) (*n* = 6), each dot in the plots represents a single animal; *p* values were determined using one‐way ANOVA with Tukey post hoc test, whereas the comparison between the control and experimental groups was determined using one‐way ANOVA with Dunnett T post hoc test; **p < 0.01, ***p < 0.001, ****p < 0.0001. C) Fluorescent images of PIEZO2 and nerves (PGP9.5) for the No Gel, pGel/M‐NIR, and MpGel‐NIR treated groups. D) Scheme of skin wound repair with implications for adnexal restoration, vascular and neural network regeneration, and functional recovery modulation by the photothermal controlled‐release immuno‐educating nanoplatform.

Nociceptive nerves enable the human body to sense noxious stimuli and provide feedback to protect the extremities from injury. However, the loss of mechanical nociception is one of the most prevalent complications of diabetes, and its recovery is one of the most challenging tasks associated with IDU repair. The recovery of mechanical perception was based on the excellent recovery of neural structure. To explore whether MpGel‐NIR had the potential to improve the recovery of mechanical nociception, mechanical pain threshold testing was conducted on mice with IDU using von Frey filaments following treatment for 4 weeks (**Figure** **8**A). The MpGel‐NIR group had a much lower mechanical pain threshold than the other experimental groups (Figure 8B and Figure S18, Supporting Information), indicating a significantly improved mechanical nociception in mice. To understand if the regenerated neural networks were responsible for mechanical nociception, we performed the von Frey test on the MpGel‐NIR‐endo groups. If the neuronal networks we identified were in charge of functional recovery, then the endostatin‐induced failure of angiogenesis (Figure 6A–C) leading to almost no regeneration of neural networks (Figures 6C and 7B) should have also inhibited mechanical nociception recovery. Thus, MpGel‐NIR‐endo animals had a significantly higher mechanical threshold compared to healthy mice (Figure 8B), suggesting that the identified neural networks were implicated in the recovery of mechanical nociception, further validating the essential role of angiogenesis in the neural system. Further, to explore the link between the restored mechanical nociception and the neural structures, we co‐stained the piezo‐type mechanosensitive ion channel component 2 (PIEZO2) protein and PGP 9.5. Patients with diabetic peripheral neuropathy have been shown to be deficient in the mechanical stimuli‐sensitive ion channel PIEZO2, which is reported to partially mediate mechanical nociception, a mechanism to prevent further injury.^[^ ^75^ ^]^ Here, the co‐staining fluorescence images revealed that PIEZO2 expression was enhanced in MpGel‐NIR and highly overlapped with the position of PGP9.5 compared with No Gel and pGel/M‐NIR groups (Figure 8C), suggesting that the regenerated peripheral nerves have a potentially normal protective function with a heightened sensitivity to pain, touch, and vibration rather than peripheral neuropathy. Hence, the nerve regenerated by MpGel‐NIR was not pathological but rather capable of expressing PIEZO2 and experiencing mechanical nociception. Therefore, it compensated for the deficiency of external mechanical injury perception and provided a potentially effective strategy for preventing the recurrence of diabetic ulcers.

### 2.7 Major Organ Histology, Routine Blood Tests, and Biochemical Tests

The major organs (heart, liver, spleen, lung, and kidney) of mice in all groups were taken 28 days after operation, and H&E staining was performed for further pathological examination. The results showed that no obvious acute or chronic pathological toxicity of major organs was observed in all groups including MpGel‐NIR, indicating that the histological abnormalities of major organs of MpGel‐NIR were negligible (Figure S19, Supporting Information). On the 28th day after operation, peripheral blood was taken from mice in all treatment groups for routine blood tests and blood biochemical tests. Routine blood tests indicators include red blood cell, hemoglobin (HGB), mean corpuscular volume, mean corpuscular HGB, mean corpuscular HGB concentration, platelet count/blood platelet count, alanine aminotransfer, aspartate aminotransfer, creatin, and blood urea nitrogen. The routine blood tests results showed that the levels of all indicators in MpGel‐NIR group were equivalent to those of normal mice, indicating that MpGel‐NIR did not cause changes in blood routine indicators (Figure S20A, Supporting Information). Furthermore, blood biochemical tests results showed that No Gel, Empty Gel, pGel, and Gel/M had higher white blood cell (WBC) and Gran levels, followed by pGel/M, MpGel, pGel‐NIR, pGel‐M‐NIR, and MpGel‐NIR‐endo (Figure S20B, Supporting Information). The MpGel‐NIR group was equivalent to the healthy mice, with the lowest WBC and Gran levels (Figure S20B, Supporting Information). These results showed that MpGel‐NIR group reduced inflammatory cells in the blood, which was consistent with the results of local histological inflammation detection.

## 3 Conclusion

IDU treatment involves a synergistic and effective strategy to resist bacteria, release chronic inflammation, stimulate regenerative development, and achieve mechanical nociception, breaking the pathological vicious circle. Our research demonstrates that the photothermal controlled‐release hydrogel nanoplatform (MpGel) exhibits a switched‐controlled release of Mup by NIR on–off, providing a pro‐regenerative environment and excellent antibacterial effects. Furthermore, pGO promotes angiogenesis by recruiting Trem2^+^ macrophages (Figure 8D). Notably, the regenerated vascular bed was sufficiently mature to germinate physically associated neural networks (Figure 8D). While, blocking the VEGF‐eNOS signaling axis, maturation of blood vessels, and formation of vascular networks by incorporating endostatin, inhibited the formation of the neural networks further, which indicated that blood vessels establish a reparative neurovascular niche and lead to neural network reconstruction in the wound area, similar to tissue development under normal physiological conditions. Hence, the antimicrobial and immunomodulatory actions of the hydrogel nanoplatform promote the development of vascular, accompanied by neuronal networks. The behavioral test results of mice based on von Frey test showed that MpGel‐NIR restored the mechanical nociception of mice. MpGel‐NIR‐endo animals had a significantly higher mechanical threshold compared to healthy mice, suggesting that the identified neural networks were implicated in the recovery of mechanical nociception. The co‐staining fluorescence images revealed that the expression of PIEZO2, mechanical stimuli‐sensitive ion channel, was significantly enhanced in MpGel‐NIR and highly overlapped with the position of PGP9.5. Hence, the nerve regenerated by MpGel‐NIR was not pathological but rather capable of expressing PIEZO2 and experiencing mechanical nociception. In all, a full‐stage strategy from antibacterial, immune regulation, angiogenesis, and neurogenesis, to the recovery of mechanical nociception, an indispensable neural function of skin, is introduced to IDU treatment, which opened up a comprehensive new way for the treatment of IDU.

## Conflict of Interest

The authors declare no conflict of interest.

## Supporting information

Supporting Information

Supplemental Video 1

## Data Availability

The data that support the findings of this study are available from the corresponding author upon reasonable request.
