# Supplementary material for: Photothermal Controlled‐Release Immunomodulatory Nanoplatform for Restoring Nerve Structure and Mechanical Nociception in Infectious Diabetic Ulcers
Source: Adv Sci (Weinh). 2023 May 6;10(20):2300339. doi: 10.1002/advs.202300339 (PMC10369251; doi:10.1002/advs.202300339)
Supplement: Supplementary file 1 — Supporting Information [file ADVS-10-2300339-s001.pdf]

ADVANCED SCIENCE
Open Access

Supporting Information

for *Adv. Sci.*, DOI 10.1002/advs.202300339

Photothermal Controlled-Release Immunomodulatory Nanoplatform for Restoring Nerve Structure and Mechanical Nociception in Infectious Diabetic Ulcers

Le Jiang, Xiangyi Wu, Yifan Wang, Chunlin Liu, Yixian Wu, Jingyun Wang, Nan Xu, Zhijun He, Shuqin Wang, Hao Zhang, Xiumei Wang, Xiong Lu, Qian Tan and Xiaodan Sun**

Supporting Information for

Photothermal controlled-release immunomodulatory nanoplatform for restoring nerve structure and mechanical nociception in infectious diabetic ulcers

Le Jiang [1,3]†, Xiangyi Wu [2]†, Yifan Wang [1,3]†, Chunlin Liu [1,3], Yixian Wu [1,3], Jingyun Wang [1,3], Nan Xu [1,3], Zhijun He [1,3], Shuqin Wang [2], Hao Zhang [2], Xiumei Wang [1,3], Xiong Lu [4], Qian Tan [2]*, and Xiaodan Sun [1,3]*

1 State Key Laboratory of New Ceramics and Fine Processing, School of Materials Science and Engineering, Tsinghua University, Beijing 100084, People's Republic of China.

2 Department of Burns and Plastic Surgery, Nanjing Drum Tower Hospital, the Affiliated Hospital of Nanjing University Medical School, No. 321, Zhongshan Road, Nanjing, Jiangsu, China. 210008

3 Key Laboratory of Advanced Materials of Ministry of Education of China, School of Materials Science and Engineering, Tsinghua University, Beijing 100084, People's Republic of China.

4 Key Lab of Advanced Technologies of Materials, Ministry of Education, School of Materials Science and Engineering, Southwest Jiaotong University, Sichuan, Chengdu, 610031, China

† These authors contribute equally to the paper

* Correspondence: Prof. Qian Tan, smmutanqian@sina.com

Prof. Xiaodan Sun, sunxiaodan@tsinghua.edu.cn

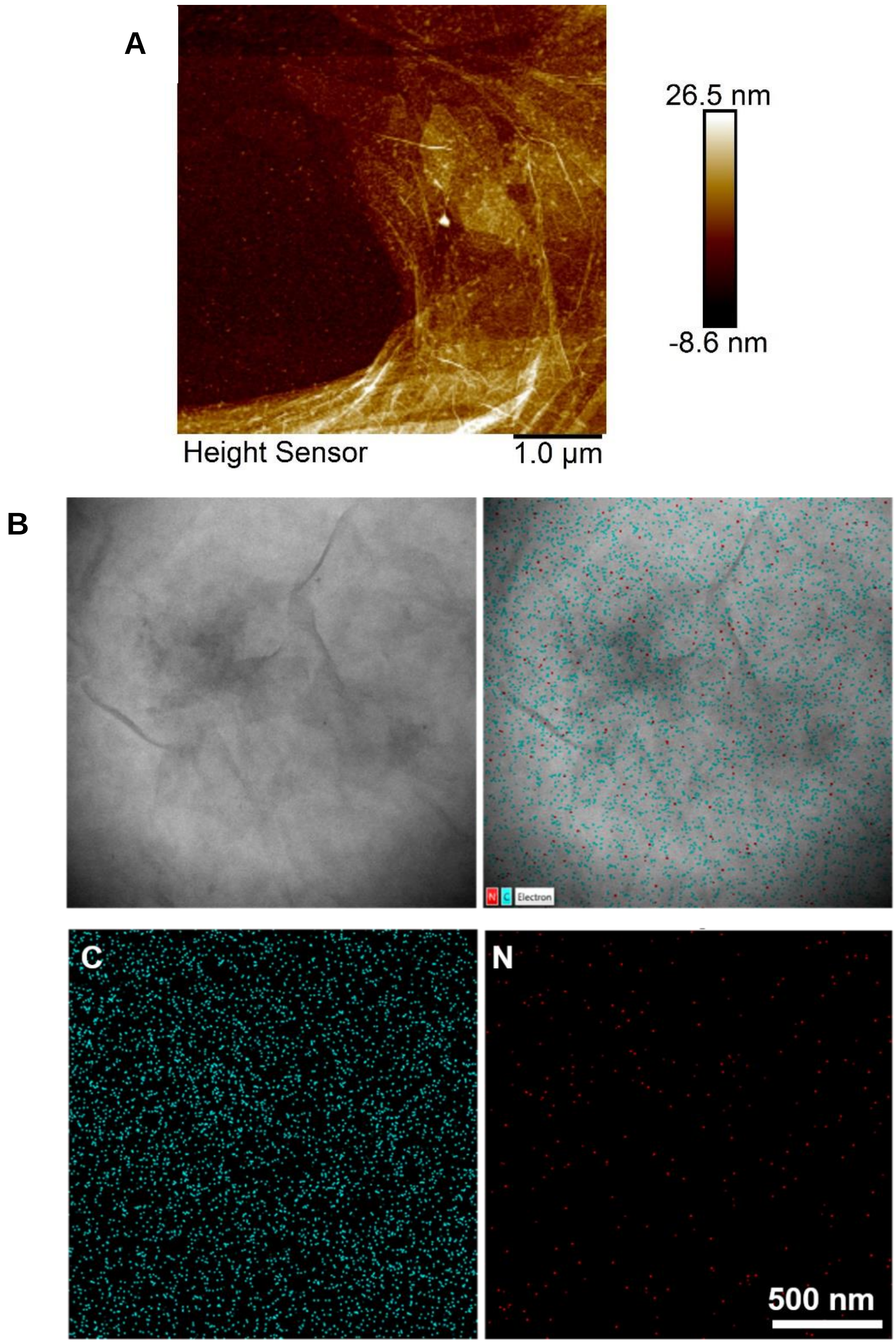

Figure S1. Morphology and composition of pGO (A) Atomic Force Microscope scanning images of pGO (B) Transmission electron microscope images of pGO and carbon and nitrogen element distribution on a slice of pGO characterized by Energy Dispersive Spectroscopy

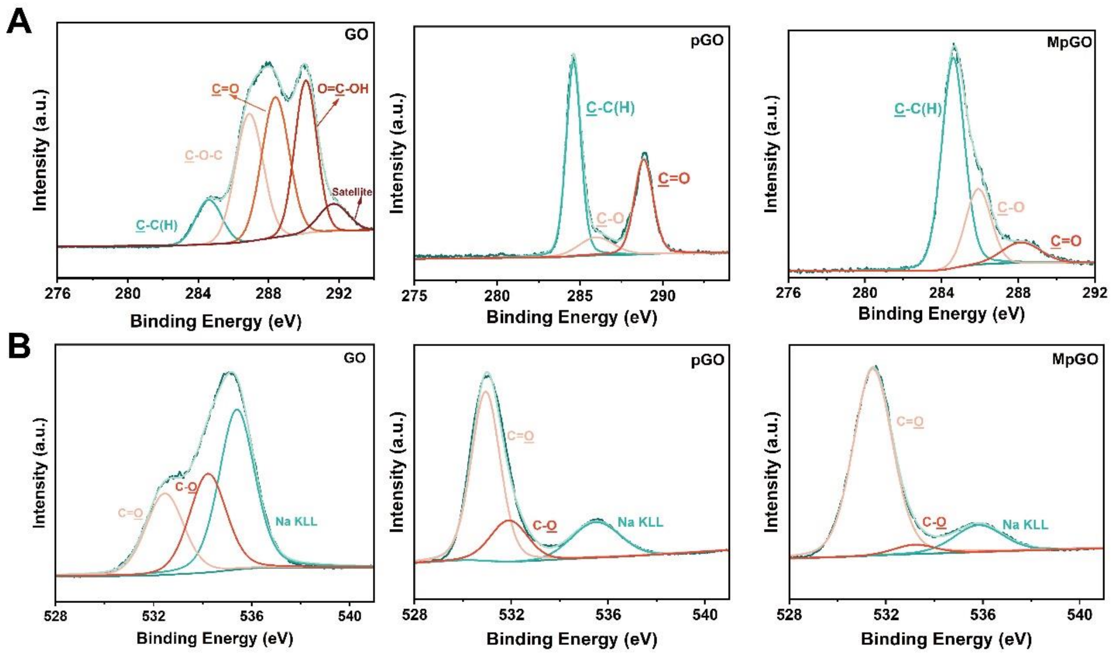

Figure S2. High-resolution XPS spectra of GO, pGO and MpGO, (A)C 1s, (B) O 1s

Table S1. Curve fitting results for the XPS C 1s spectra of GO, pGO and MpGO

Functional groups	Binding Energy (eV)	GO (%)	pGO (%)	MpGO (%)
C-C (H)	284.6	9.6	54.6	65.5
C-O-C	286.4	29.7	11.9	24.6
C=O	288.5	32.2	33.6	9.9
HO-C=O	290.1	28.4	0	0

Peak at 291.1 eV in figure S1(A) is the satellite peak of extended delocalised electrons.

Table S2. Curve fitting results for the XPS O 1s spectra of GO, pGO and MpGO

Functional groups	Binding Energy (eV)	GO (%)	pGO (%)	MpGO (%)
C-O	533.1	45.8	23.1	5.0
C=O	531.8	54.2	76.9	95.0

Peak at 535 eV in figure S1(B) is the sodium Auger peak, and this is also confirmed by the wide XPS spectrum, (0~1200 eV, not shown here), where peaks at 1071 eV and 497 eV are displayed. The ratio between C-O and C=O calculated by O 1s spectra is roughly consistent with that calculated by C 1s for GO and pGO, while for MpGO they are not consistent, potentially because the concentration of mupirocin is relatively low, therefore, when X-ray irradiates the area containing mupirocin the C-O proportion is higher, otherwise C=O proportion higher.

A

B

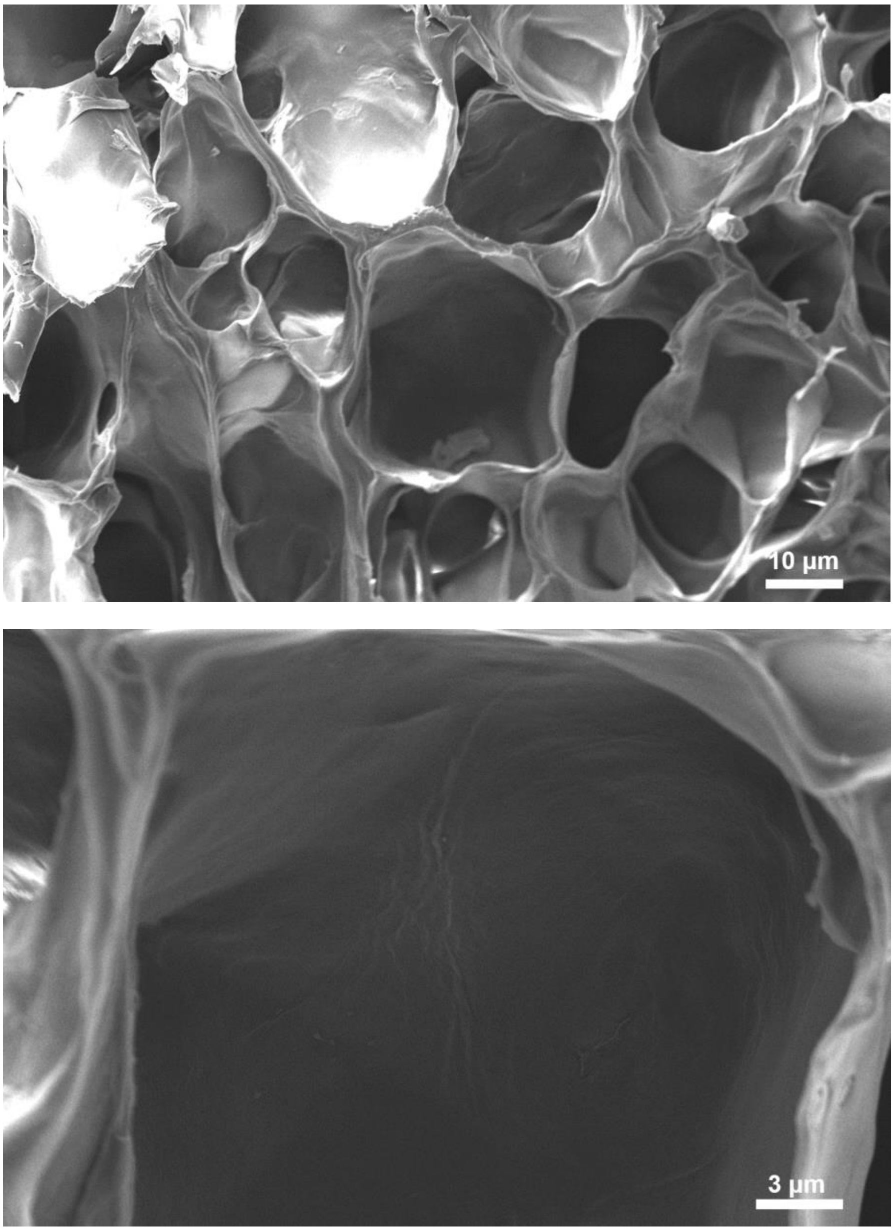

Figure S3. Porous structure of pGel at scale of 10 μm (A) and 3 μm (B) characterized by scanning electron microscope.

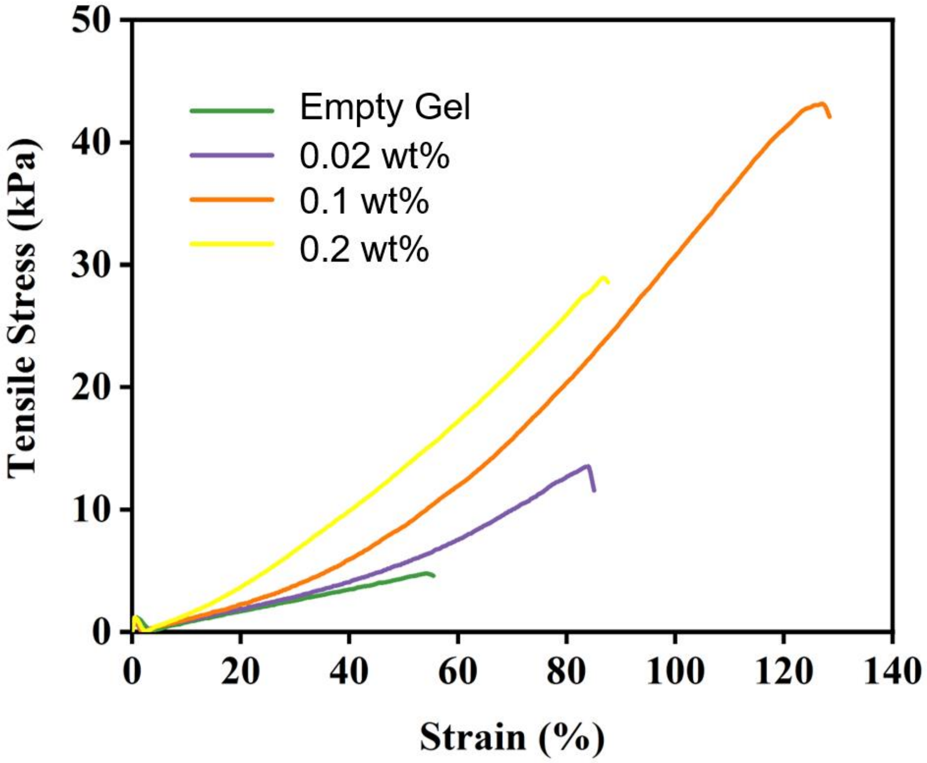

Figure S4. Tensile strength-strain curve of hydrogels added with 0, 0.02 wt%, 0.1wt%, 0.2 wt% of pGO.

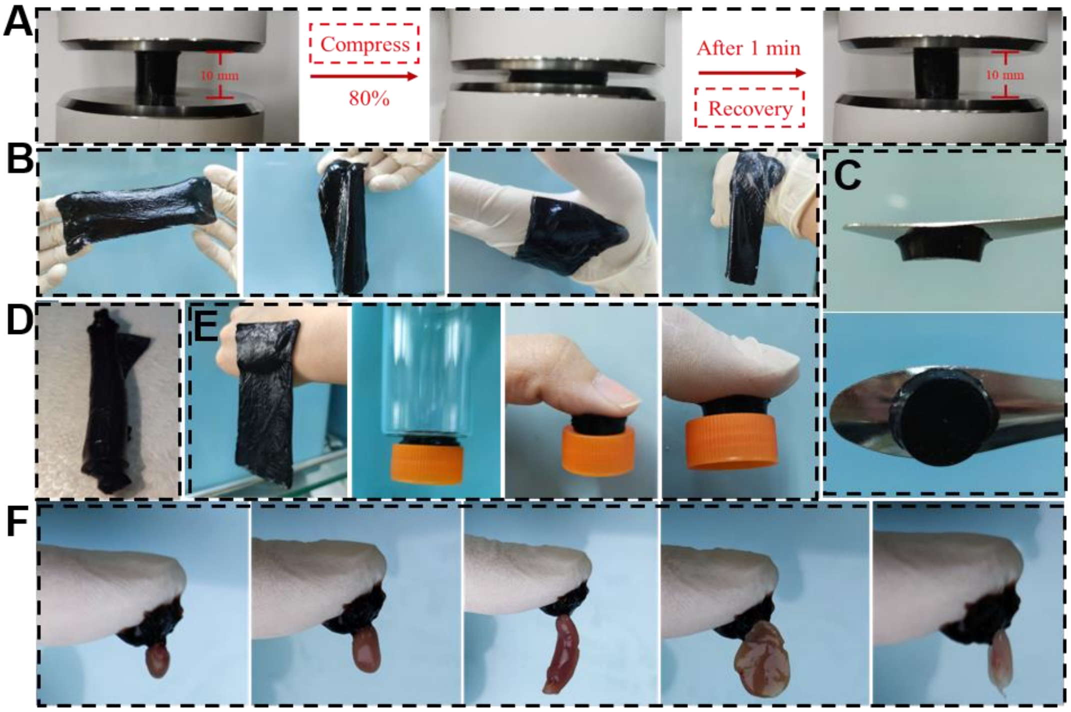

Figure S5. the elasticity and adhesivity of the MpGel platform. (A) Compressive elasticity testing of the hydrogel. (B) Flexibility, ductility and adhesivity to the glove. (C) Adhesivity to the lab spoon. (D) Flexibility. (E) Adhesivity to the skin, plastic and glass, and the adhesive force overcoming weight. (F) Adhesivity to the heart, liver, spleen, lung and kidney from mice.

A

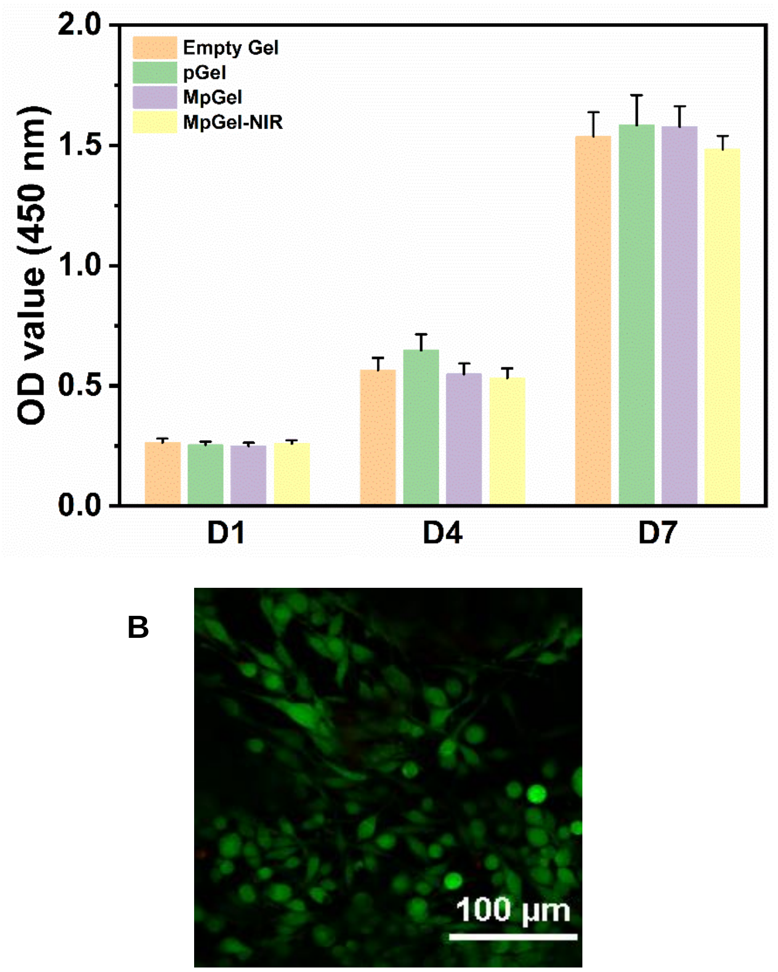

Figure S6. (A) CCK8 testing on L929 cells cultured in the situation of Empty Gel, pGel, MpGel and MpGel-NIR; (B) Live-death test on Schwann Cells cultured on pGel.

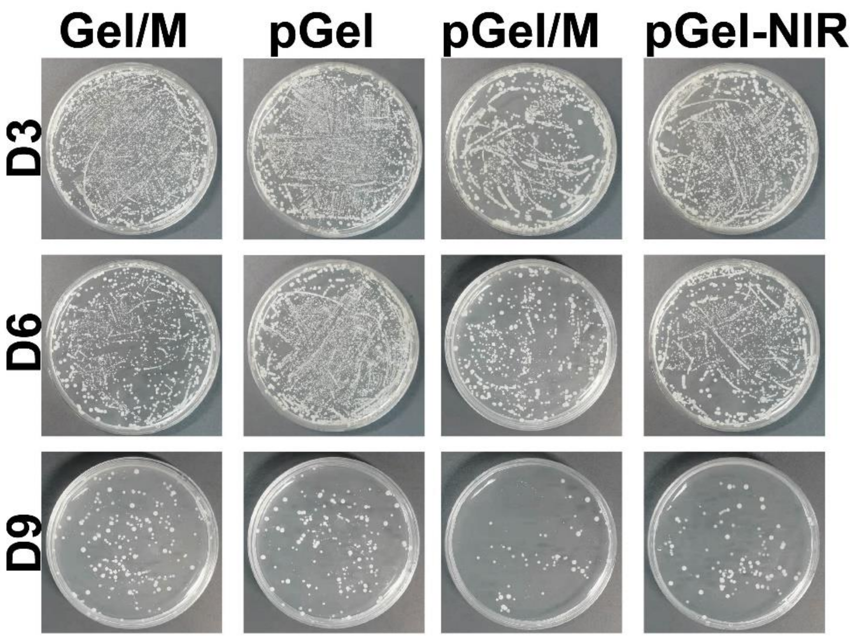

Figure S7. Photo of antibiotic activity *in vivo* on day 3rd, 6th, and 9th of Gel/M, pGel, pGel/M, pGel-NIR treated groups;

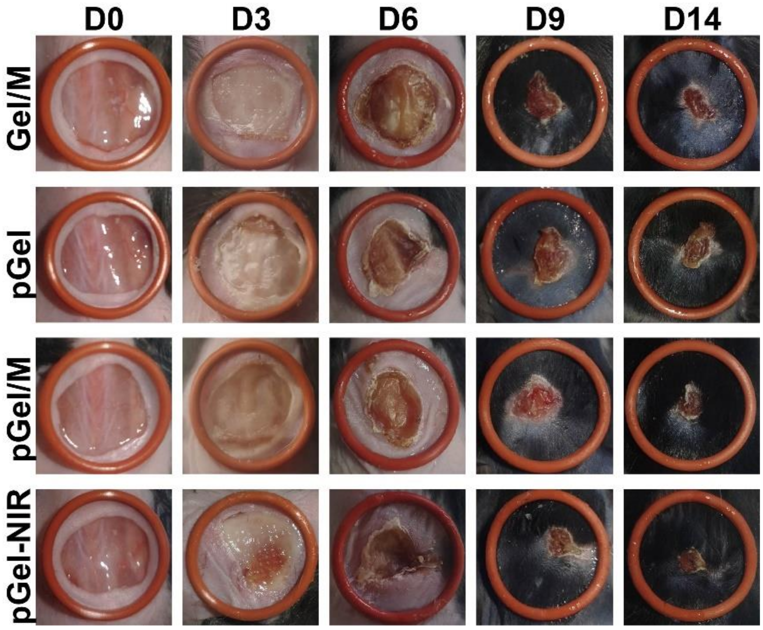

Figure S8. Photo of skin wound area on day 0, 3rd, 6th, 9th and 14th of Gel/M, pGel, pGel/M, pGel-NIR treated groups.

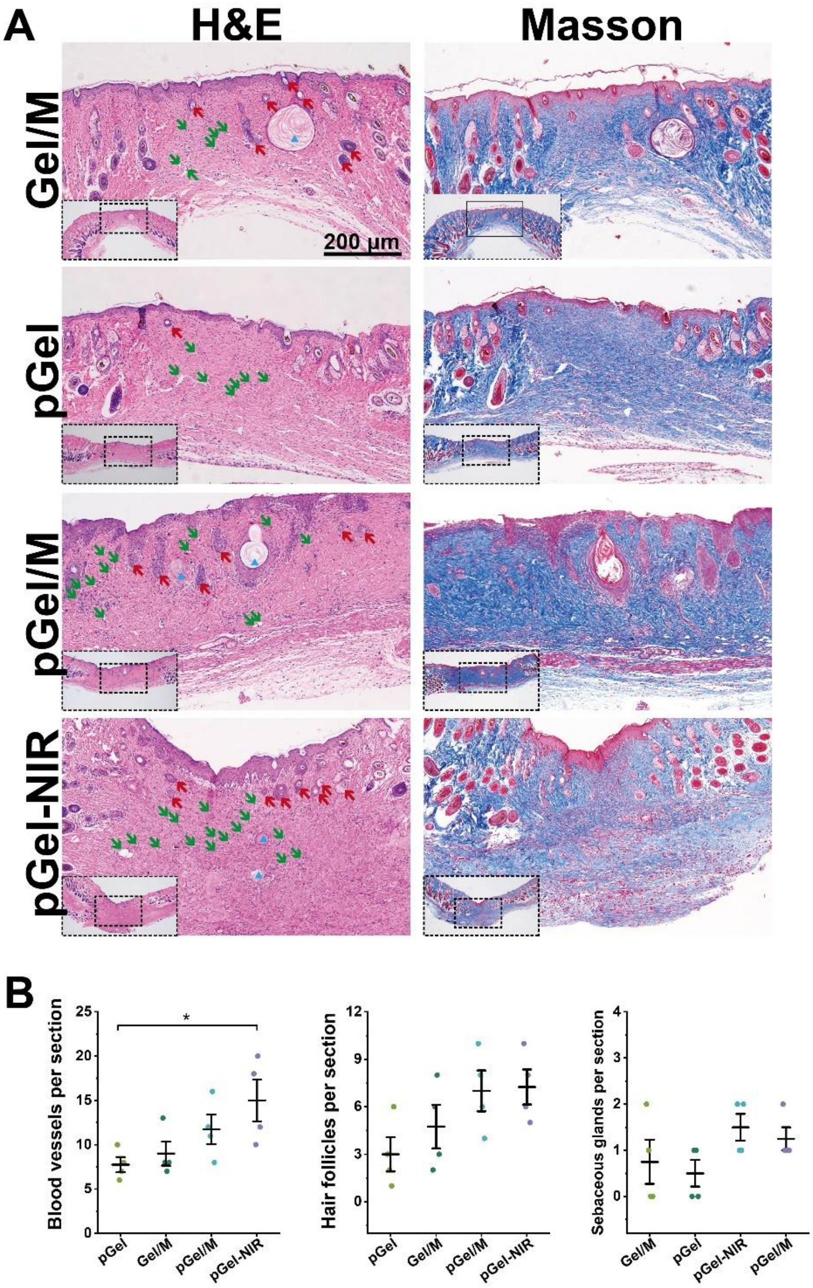

Figure S9. (A) H&E and masson staining of the wound skin of Gel/M, pGel, pGel/M, pGel-NIR treated groups on day 14th (blood vessels, hair follicles and sebaceous glands are indicated by green arrows, red arrows and blue triangles respectively). (B) The number of blood vessels, hair follicles and sebaceous glands per section (n = 4) and quantification of the collage fraction. Each dot in the plots representing one animal and P values of blood vessels, hair follicles in (B) were determined by one-way ANOVA with Tukey post hoc test while for sebaceous glands were determined by nonparametric test using Kruskal–Wallis and all pairwise for multiple comparison；*P<0.05.

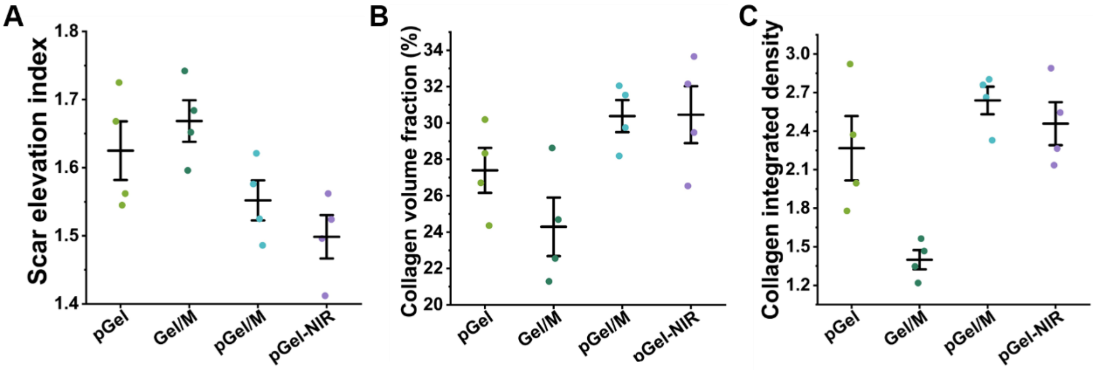

Figure S10. (A) Scar elevation index (B) collagen volume fraction and (C) collagen integrated density of pGel, Gel/M, pGel/M and pGel-NIR.

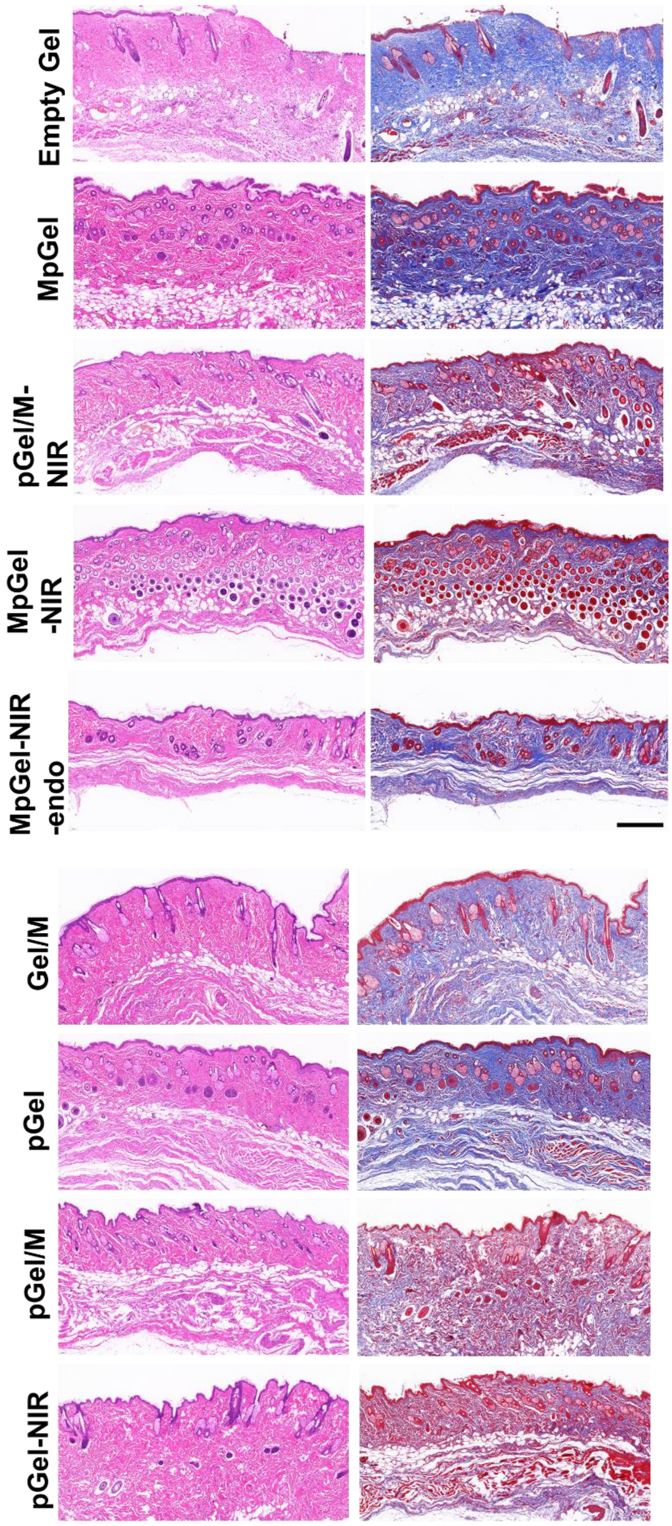

Figure S11. H&E (the left row) and Masson staining (the right row) of the wound skin of Empth Gel, MpGel, pGel/M-NIR, MpGel-NIR, MpGel-NIR-enod, Gel/M, pGel, pGel/M, pGel-NIR treated groups on day 28th, the scale bar representing 300μm.

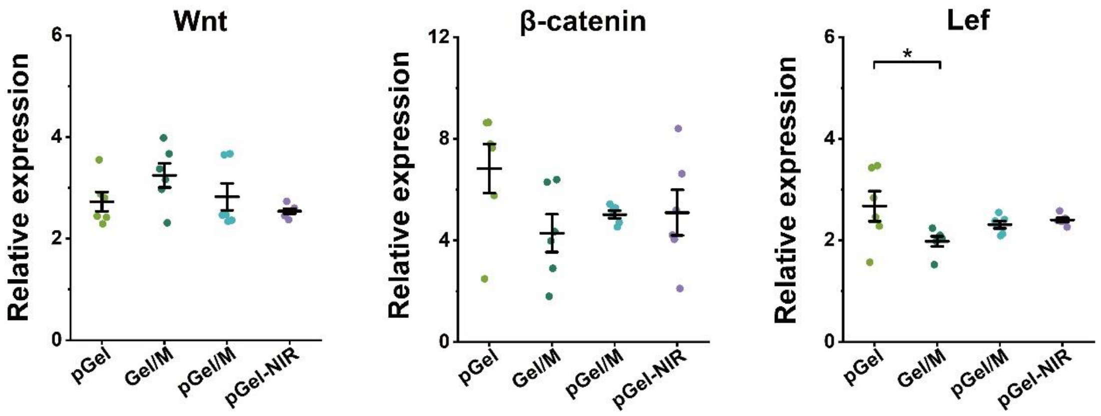

Figure S12. Relative mRNA expression of Wnt, β-catenin, and Lef, (n = 6), each dot in the plots representing one animal and P values of β-catenin were determined by one-way ANOVA with a Tukey's post-hoc test, while for Wnt and Lef the P values were determined by nonparametric test using Kruskal–Wallis and all pairwise for multiple comparison, *P<0.05.

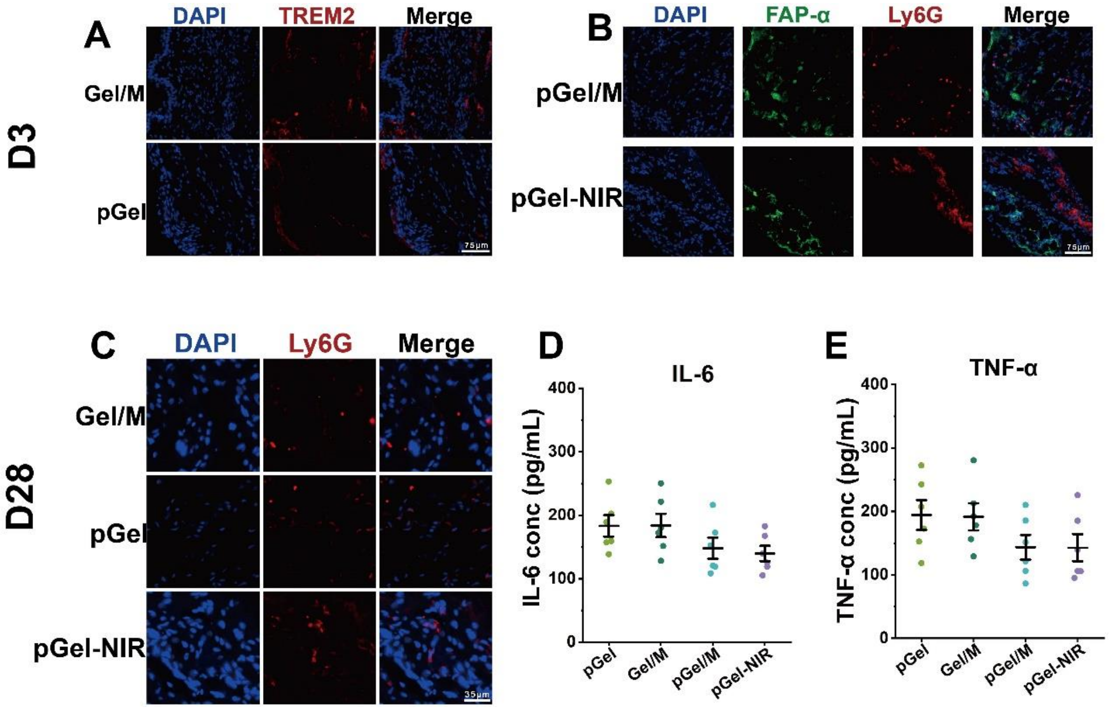

Figure S13. (A)Fluorescent images of Trem2^{+} macrophages (TREM2) of Gel/M, pGel treated groups on day 3rd. (B) Fluorescent images of FAP-α+ fibroblasts (FAP-α) and neutrophils (Ly6G) of pGel/M, pGel-NIR treated groups on day 3rd. (C) Fluorescent images of neutrophils (Ly6G) of Gel/M, pGel, pGel-NIR treated groups on day 28th. (D-E) **(D, E)** ELISA results of the expression concentration of IL-6 (D) and TNF-α(E).

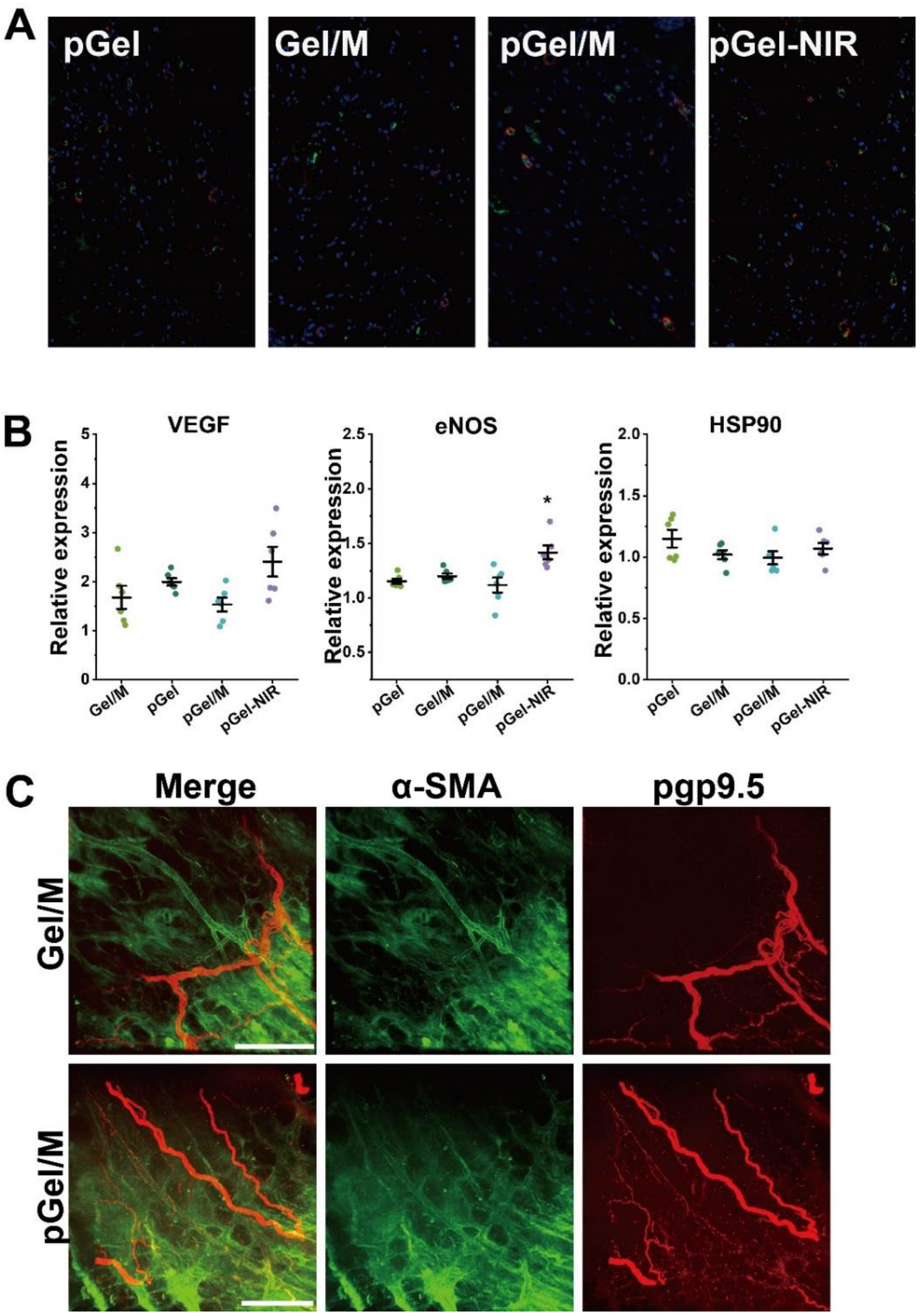

Figure S14. (A) Fluorescence images of sections with endothelial cells (CD31) in green and blood vessels (α-SMA) in red. (B) Plots of relative mRNA expression of VEGF, eNOS, and HSP90, (n = 6); each dot in the plots representing one animal and P values of eNOS in were determined by one-way ANOVA with Tukey post hoc test while the others were determined by nonparametric test using Kruskal–Wallis and all pairwise for multiple comparison；*P<0.05. (C) Fluorescent images of cleared wound skin tissue with blood vessels (α-SMA) in green and nerves (PGP9.5) in red after treated with Gel/M, pGel/M.

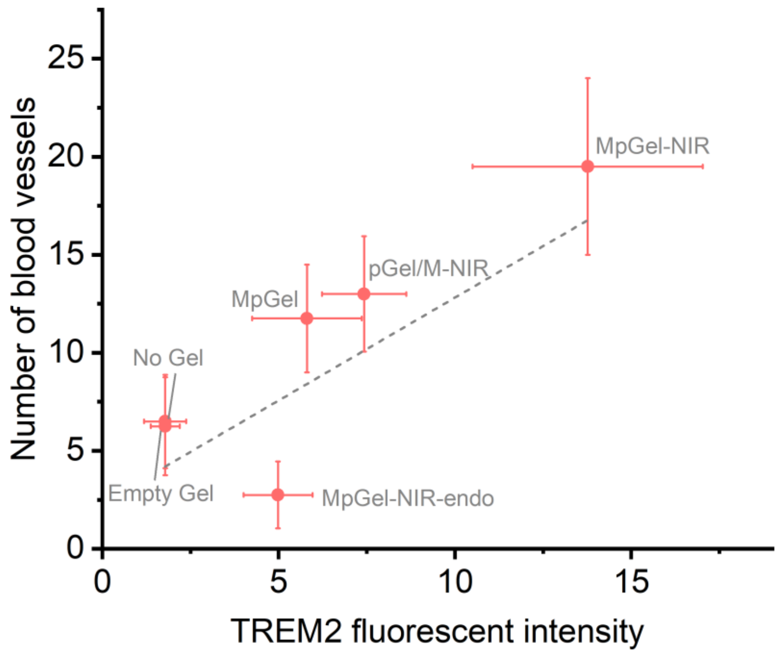

Figure S15 A rough Pearson correlation analysis is made, where a point represents a pair of data (the average number of blood vessels and the average intensity of TREM2 fluorescence) from the same experiment group (n = 4, here, take one group of mice as the statistical individual). The Pearson correlation coefficient r = 0.864, and P value is less than 0.05 (0.027), indicating a high correlation between the number of blood vessels and intensity of TREM2 fluorescence. Both of the variations obey normal distribution, and the analysis is conducted by IBM SPSS Statistics 22.0.

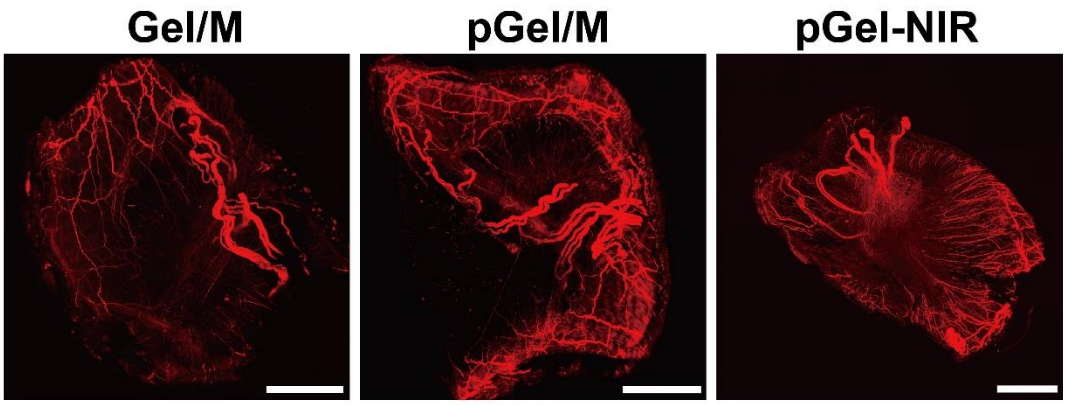

Figure S16. Fluorescent images of bulk wound skin tissues with nerves system (PGP9.5) in red for Gel/M, pGel/M and pGel-NIR treated groups.

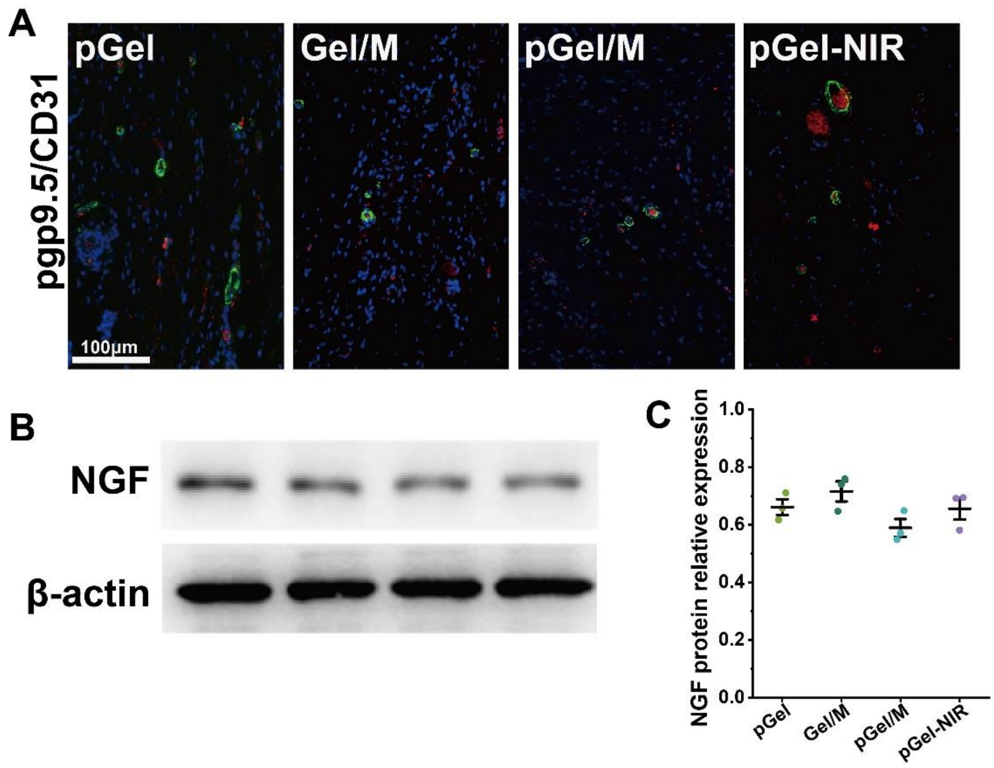

Figure S17. (A) Fluorescent images of cleared wound skin tissue with blood vessels (α-SMA) in green and nerves (PGP9.5) in red after treated with Gel/M, pGel/M. (B) Fluorescence images of sections with endothelial cells (CD31) in green and nerves (PGP9.5) in red for Gel/M, pGel, pGel/M, pGel-NIR treated groups. (C, D) Western-blotting of NGF for Gel/M, pGel, pGel/M, pGel-NIR treated groups (C) and the quantification of relative expression (n = 3) (D), and statistical analysis were determined by one-way ANOVA with Tukey post hoc test.

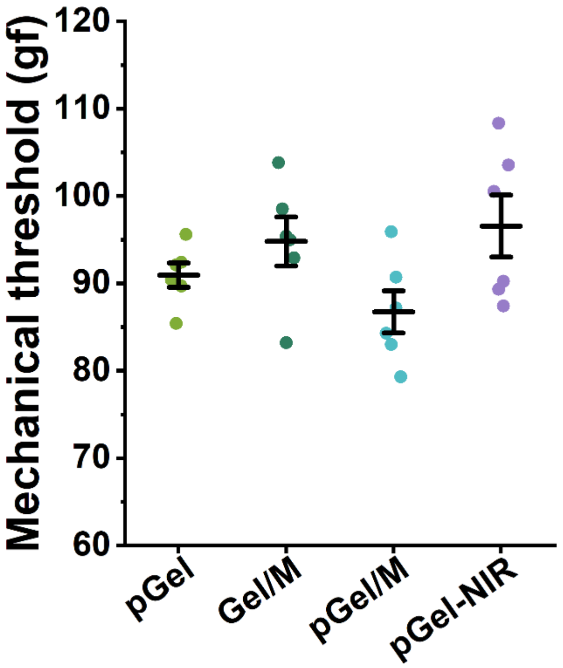

Figure S18 Quantification of mechanical threshold (n = 6), each dot in the plots representing one animal and statistical analysis were conducted by one-way ANOVA with Tukey post hoc test.

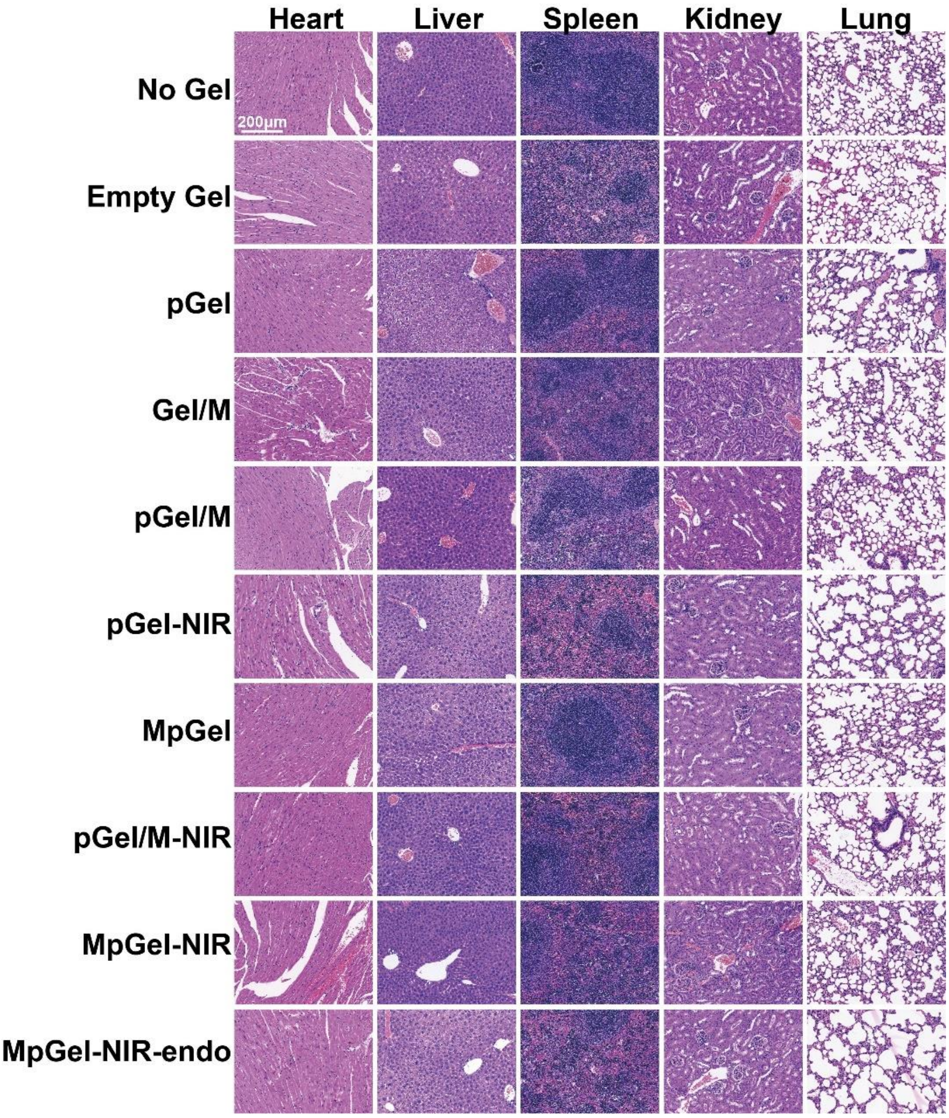

Figure S19. Biosafety assay of the hydrogels in vivo through H&E staining of heart, liver, spleen, kidney and lung harvested from mice treated with No Gel, Empty Ge, pGel, Gel/M, pGel/M, pGel-NIR, MpGel, pGel/M-NIR, MpGel-NIR and MpGel-NIR-endo.

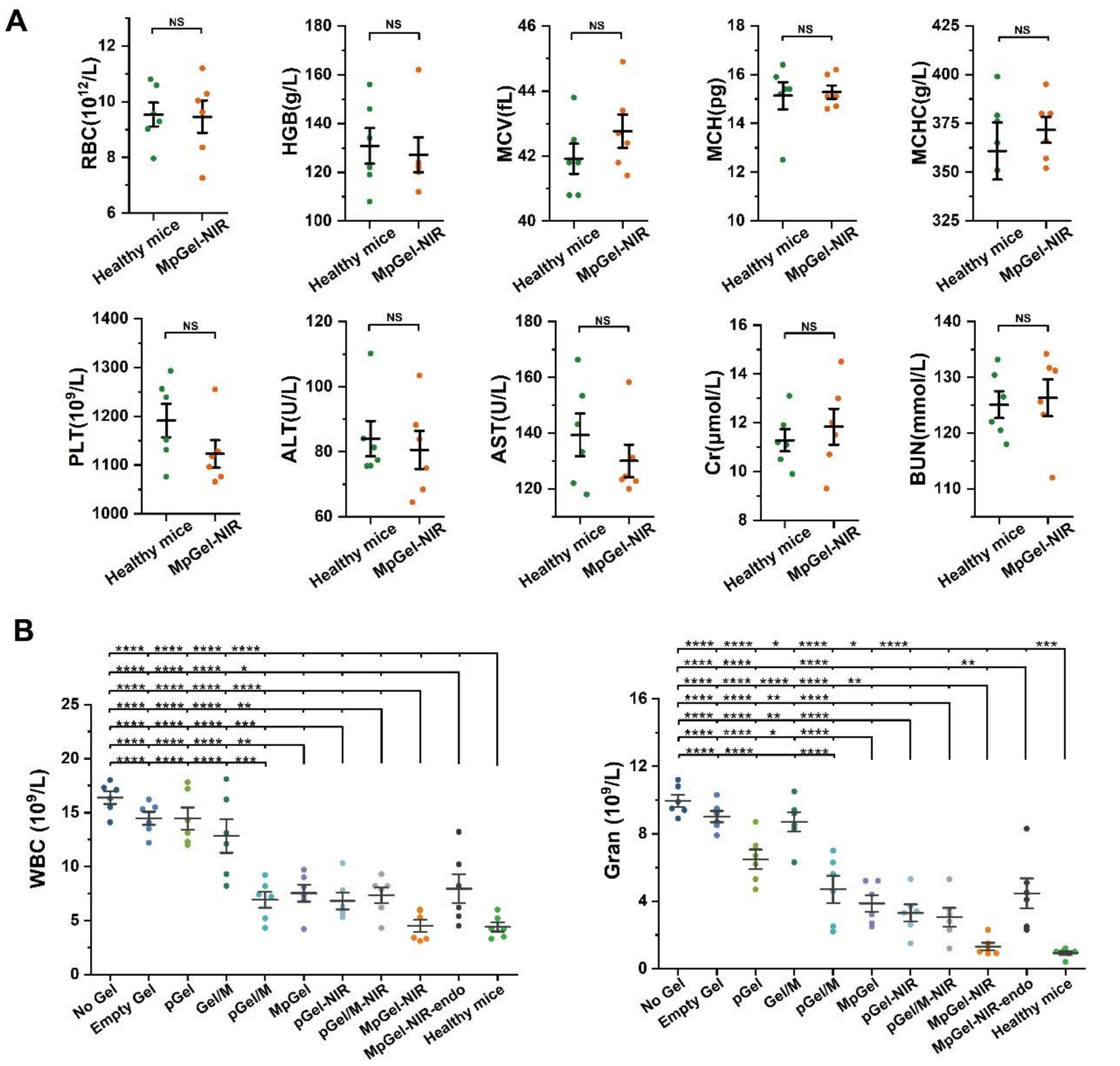

Figure S20. (A) routine blood test for red blood cell (RBC), hemoglobin (HGB), mean corpuscular volum (MCV), mean corpuscular hemoglobin (MCH), mean corpuscular hemoglobin concentration (MCHC), platelet count/blood platelet count (PLT), alanine aminotransferase (ALT), aspartate aminotransferase (AST), creatinine (Cr) and blood urea nitrogen (BUN) (B) Blood biochemical examination of white blood cell (WBC) and Gran

Methods

Syntheses of GO, pGO, MpGO

GO was prepared by the Hummers method [1]. Under an ice water bath, graphite powder (10 g, sigma, USA) was added to 230 ml H_2SO_4 and stirred continuously for half an hour. Keeping the ice water bath, $KMnO_4$ (50g) was slowly added to the solution. Subsequently, the solution was stirred at 37 °C for 30 minutes and at 25 °C for 24 hours. Deionized water (900 ml) was then slowly added to the mixture. The solution reaction lasted for 30 minutes. The reaction mixture was cooled to room temperature. Finally, 60ml H_2O_2 was added to the solution to terminate the reaction. The mixture was filtered using filter paper and washed three times with deionized water and absolute ethanol. The mixture was centrifuged at 3000 rpm for 8 min; The resulting supernatant is the GO solution.

pGO was prepared according to our previous method [1]. Briefly, DA (0.02g) was added to the GO solution (10ml, 0.2wt%) with stirring at room temperature, and the pH of the solution was adjusted to 8.5. After 2 hours, the mixture was centrifuged (10000rpm, 5 minutes) to obtain pGO.

50 mg mupirocin was added to 20 mL pGO solution, and then perform magnetic stirring for 24h followed by centrifugation. The supernatant was removed and washed with deionized water for three times to obtain MpGO.

Characterization of pGO

The morphology of nanomaterials was obtained by a JEM-2100F Field emission transmission electron microscope (TEM, 200 kV) and atomic force microscope (AFM). X-ray diffraction (XRD) was measured on a D/max-2550 XRD system (parameters: Cu Kα, λ = 1.54 Å, 40 mA, and 40 kV). Hydrated particle size was obtained by Malvern nanosize. X-ray photoelectron spectroscopy (XPS) spectrum was conducted on an 250XI (Thermal Scientific, US). Raman spectrum was recorded by LabRAM HR Evolution (Horiba, France).

Preparation of hydrogels

Firstly, 1 g acrylamide (AM, purchased from Sinopharm Chemical Reagent Co., Ltd), and 0.04 g dopamine acrylamide (DAm, prepared as reported before [2]), 0g/0.02g(0.02wt%)/0.1g(0.1wt%)/0.2g(0.2wt%) pGO and 0.6g chitosan (CS, purchased from Sinopharm Chemical Reagent Co., Ltd) was added into the 10 mL

ddH_2O, stirring with a magnetic stirrer in an ice bath. Secondly, 1 mL N,N,N',N'-tetramethylethylenediamine (TEMED, purchased from Sigma-Aldrich) with the concentration of 0.5 M, 1.74 g acrylic acid (AA, purchased from Sigma-Aldrich), and 1 mL 0.5 M ammonium persulfate (APS, purchased from Sinopharm Chemical Reagent Co., Ltd) was added into the above solution in turn, followed by transferring to the mold and gelling at 60 °C for 4 hours. MpGel is obtained by adding 0.1 wt% MpGO, while pGel/M is obtained by adding equal amounts of Mup and pGO.

Characterization of hydrogels

The morphology of hydrogels was obtained by high resolution Zeiss field emission scanning electron microscopy. The chemical structure of hydrogel is synthesized by Fourier transform infrared spectrometer (FTIR, Nicolet iS50, Thermo Fisher Scientific, USA) in the wavenumber range of 4000-400 cm^{-1} and a resolution of 0.48 cm^{-1}. Tensile strength-strain curve obtained by universal mechanical testing machine.

NIR light-triggered Mup release from MpGel and pGel/M

Release of Mup from MpGel and pGel/M was determined in DI water. 1 mL of DI water (above) was placed in a 24-well cell culture plate containing 0.5 mL of MpGel or pGel/M (below). MpGel or pGel/M was irradiated using an 808 nm NIR laser (0.5 W/cm^2) for 10 min followed by an interval of 30 min and the procedure was repeated three times. Then, a liquid chromatography-mass spectrometry system (AB Qtrap 6500) was used to analyze the above solution to determine the release amount of Mup. In comparison, MpGel or pGel/M without NIR light irradiation was set as a control.

CCK8 assay

Divide the experiment into four groups：Gel, pGel, MpGel, MpGel-NIR(3min). L929, a Mouse fibroblast cell line, with an initial density of 2×10^5 /mL were seeded on hydrogels and cultured in a 5% CO2 incubator at 37 °C. The cell culture medium was replaced every other day. Cell proliferation on the hydrogels was evaluated by cell counting kit-8 reagent (CCK8, Dojindo Molecular Technologies) after 1, 4, 7 days of culture.

Live-death test on Schwann Cells cultured on pGel.

After 3 days of culture in the growth medium, the Schwann Cells were rinsed twice with 1×PBS pre-warmed at 37°C.The cytotoxic effect of the hydrogel was evaluated with Calcein-AM/PI (Dojindo Molecular Technologies) followed by observation with Confocal laser microscope (CLSM).

***In vivo* animal experience**

The C57BL/6 mice were obtained from Beijing Hufukang Biotechnology Co., LTD. After one week of adaptive feeding, the mice were administered STZ (100 mg/kg) with 0.01M citrate buffer as a vehicle once intraperitoneally. The random blood glucose of each mouse was monitored every two days and when it reached 11.1 mmol/l, the type I diabetes model was considered to be successfully established. Following anesthetization, one full-thickness skin wound with a diameter of 10mm was created on the dorsum and 10 μl of bacteria liquid (Staphylococcus *aureus*, 1×10^7 CFU) was applied to the wound. After that, the mice were randomly divided into ten groups (n=20) and treated with No Gel, Empty Gel, Gel/M, pGel, pGel-NIR, pGel/M, MpGel, pGel/M-NIR, MpGel-NIR, MpGel-NIR-endo respectively. In the MpGel-NIR-endo group, 100 μl endostatin (100 μg/ml) was injected subcutaneously in the peritraumatic area before the application of the treatment. Every time before sampling, all mice were euthanized by CO_2 asphyxiation. The animal experiments were performed according to the relevant ethical regulations and approved by Animal Protocol (21-SXD1) of Institutional Animal Care and Use Committee (IACUC) in Tsinghua University.

ELISA assay

To evaluate the inflammation on the 3rd day of treatment, we detected the level of IL-6 and TNF-α in the wound. Wound tissue was homogenized and the supernatant was collected for ELISA assay with commercial ELISA kits (Mouse TNF-α and IL-6 ELISA KIT, Solarbio). The experiments were carried out according to the manufacturers' instructions.

Antibacterial activity *in vivo*

To evaluate the antibiotic activity *in vivo*, the wound tissue was sampled for bacterial culture on the 3rd, 6th, 9th, and 12th day after the treatment. After overnight culture, 10

μl of the diluted bacterial solution was spread on the solid medium and incubated for 24 h. The viable bacteria were observed and recorded.

RNA extraction and Real-time quantitative PCR

To evaluate the gene expression level of VEGF, eNOS, HSP90, Wnt, β-catenin, and Lef, the RNA of diabetic wound tissue on the 14th day of treatment was extracted and examined with RT-qPCR assay. Steady Pure Universal RNA Extraction Kit (Accurate Biology) was used to extract the RNA and the Evo M-MLV RT Kit (Accurate Biology) was used for reverse transcription to reverse transcribe RNA to cDNA. Gene expression of VEGF, eNOS, HSP90, Wnt, β-catenin and Lef was determined by quantification Real-time polymerase chain reaction analysis (qRT-PCR, QuantStudio 7 Pro systems, Invitrogen, USA) with gene primers. The relative gene expression levels of ADSCs were obtained accordingly by normalizing the internal control β-actin. The experiments were carried out according to the manufacturers' *instructions*.

Histological and Immunofluorescence staining and analysis

Histological and immunofluorescence staining was conducted to evaluate the tissue regeneration of the wound. On day 12, the tissue of the wounds was embedded and sectioned. Hematoxylin and eosin were used for H&E staining and the Trichrome Stain Kit (Sigma-Aldrich) was used for Masson staining. The procedures were following the manufacturers' instructions. The skin thickness, hair follicles, blood vessels, and collagen deposition were observed and recorded under Optical Microscope (OM). In addition, the major organs of each mouse on the 28th day were collected for H&E staining and observed to exclude the biotoxicity of the materials. "D" indicates scar thickness, and "d" indicates adjacent normal skin thickness. Scar elevation index (SEI) is defined by the D/d ratio. The collagen volume fraction was determined by counting collagen area per square millimeter of the field. The collagen integrated density was determined by relative intensity of collagen compard to No Gel group. These results were obtained by statistics of iamge J.

Immunofluorescence staining was performed to evaluate the neovascularization and neurogenesis of the wounds as previous described [3]. For neovascularization evaluation, the sections of wound tissue on the 12th day were stained and the new blood vessels were labeled with CD31 and α-SMA. For neurogenesis evaluation, the

sections of wound tissue on the 28th day were stained and the emerging peripheral nerves and their accompanying vasculature were labeled with PGP9.5 and α-SMA, respectively. All the sections were observed under Confocal Microscope Laser Scanner. The vessel density was determined by counting the number of vessels per square millimeter of the field.

Western Blot

Western blot was performed as described previously [4]. On day 28, the tissue of wounds was sampled and lysed with Tissue Protein Extraction Reagent (Thermo Fisher). The expression of nerve growth factor (NGF) protein was quantified by the western blotting assay performed in the standard fashion. Anti-NGF antibody (Abcam) was used. Western blot bands were quantified with NIH ImageJ.

Toxicology analysis

To evaluate the biotoxicity of the materials, the major organs of each mouse on the 28th day were collected for H&E staining and observed under OM. In addition, the blood samples of each mouse were obtained for the hematology analysis and blood biochemistry assay.

Animal behavioral tests

Pain threshold (von Frey sensitivity) was tested with a calibrated set of von Frey filaments (Stoelting, Wood Dale, IL56). The animals were acclimated to the environment before the von Frey test for 15 minutes. Each von Frey hair was applied at the wound healing site for 3 s and tested 3 times at an interstimulus interval of 20s. The pain threshold was determined by the value of strength when mice flee quickly. The test was performed in a blinded manner, where the investigator did not know the identity of the animal and the study group.

Statistical Analysis

Data within this study were presented as mean ± SD. The sample size was $n \geq 3$. No data pre-processing method was utilized. All the statistical analysis of significance test were performed among three or more groups, which were analyzed by using one-way ANOVA or nonparametric test. If data showed normal distribution and variance homogeneity, Tukey's post hoc test would be performed; if data was normally

distributed but variance was not homogeneity, the Welch's correction would be performed followed by Games-Howell post hoc test; if the data was not normally distributed or had significant variance inhomogeneity, Kruskal-Wallis nonparametric test would be conducted instead of one-way ANOVA. A rough Pearson correlation analysis was made in Figure S15, where a point represents a pair of data (the average number of blood vessels and the average intensity of TREM2 fluorescence) from the same experiment group (n = 4, here, take one group of mice as the statistical individual). The statistical analysis was conducted by IBM SPSS Statistics 22.0. OriginPro 2023 software was also used for calculation of mean and standard deviation and for diagram (including plot and bar graph) drawing. $P < 0.05$ was considered statistically significant. n.s., no significant; $^{*}P<0.05$, $^{**}P<0.01$, $^{***}P<0.001$, and ****P<0.0001.

Reference

1. Han, L. et al. A mussel-inspired conductive, self-adhesive, and self-healable tough hydrogel as cell stimulators and implantable bioelectronics. *Small* **13**, 1601916 (2017).

2. Gang, F. et al. Robust magnetic double-network hydrogels with self-healing, mr imaging, cytocompatibility and 3d printability. *Chem. Commun.* **55**, 9801-9804 (2019).

3. Wang, Y. et al. Biomechanically-adapted immunohydrogels reconstructing myelin sheath for peripheral nerve regeneration. *Advanced Healthcare Materials* **11**, 2201596 (2022).

4. Nih, L. R., Gojgini, S., Carmichael, S. T.& Segura, T. Dual-function injectable angiogenic biomaterial for the repair of brain tissue following stroke. *Nature Materials* **17**, 642-651 (2018).
